# The Mechanisms of Water Exchange: The Regulatory Roles of Multiple Interactions in Social Wasps

**DOI:** 10.1371/journal.pone.0145560

**Published:** 2016-01-11

**Authors:** Devanshu Agrawal, Istvan Karsai

**Affiliations:** 1 Department of Mathematics and Statistics, East Tennessee State University, Johnson City, Tennessee, United States of America; 2 Department of Biological Sciences, East Tennessee State University, Johnson City, Tennessee, United States of America; Georgia State University, UNITED STATES

## Abstract

Evolutionary benefits of task fidelity and improving information acquisition via multiple transfers of materials between individuals in a task partitioned system have been shown before, but in this paper we provide a mechanistic explanation of these phenomena. Using a simple mathematical model describing the individual interactions of the wasps, we explain the functioning of the common stomach, an information center, which governs construction behavior and task change. Our central hypothesis is a symmetry between foragers who deposit water and foragers who withdraw water into and out of the common stomach. We combine this with a trade-off between acceptance and resistance to water transfer. We ultimately derive a mathematical function that relates the number of interactions that foragers complete with common stomach wasps during a foraging cycle. We use field data and additional model assumptions to calculate values of our model parameters, and we use these to explain why the fullness of the common stomach stabilizes just below 50 percent, why the average number of successful interactions between foragers and the wasps forming the common stomach is between 5 and 7, and why there is a variation in this number of interactions over time. Our explanation is that our proposed water exchange mechanism places natural bounds on the number of successful interactions possible, water exchange is set to optimize mediation of water through the common stomach, and the chance that foragers abort their task prematurely is very low.

## Introduction

Insect societies function as superorganisms, where complex patterns emerge via the vast number of interactions of many individuals [1]. In most insect societies, the workers carry out all of the duties relating to colony maintenance, including nest construction, offspring care, food collecting and cleaning [2]. The propensity to carry out different types of jobs is generally different among the workers, and this leads to division of labor and/or task partitioning in many societies. Because colonies and their environments are changing continuously, tasks need to change to accommodate for altering levels of different demands. To meet the new demands, colonies reallocate their workforce or new workers are produced and recruited [3, 4]. This colony-level flexibility in response to external and internal perturbations is an essential feature of colony-level performance of these societies [5, 6, 7].

Cooperative phenomena such as organization of workforce may emerge at colony level from simple behavioral rules and local interactions of the individuals [8, 9, 10, 1]. While the individual behavioral repertoire of a single individual might look very limited and somewhat random in nature, a vast number of interactions among these individuals leads to complex, adaptive and very efficient colony-level performance. The nest of social wasps, for example, is much larger than what a single individual could construct. These structures can reach considerable complexity, size and lifespan [11, 12], and yet these nest structures (for example, in *Polistes* wasps) are built by simple self-organizing processes [13, 14, 15, 16, 17, 18]. The construction of the nest also depends on the colony size: Small colonies mainly use jack-of-all-trades workers, and large colonies commonly use a mix of several types of highly specialized workers. As the colony size increases, the importance of interactions among the workers seems to increase as well, and a given job is carried out by parallel processing and task partition [7].

When an insect society uses task partition, the work is partitioned into subtasks that are connected (in most cases) sequentially [19, 20]. In the case of humans, the bucket brigade is one of the best known examples of this kind of work organization, where the water is passed from the collectors to the users via transporters [21]. The assignments of workers to the different subtasks could be permanent through strong specialization or dynamic where task switching poses a decision at individual level [22]. These decisions can commonly be considered NP-hard problems and cannot be easily solved even by approximation [23]. We also know very little on the actual mechanisms involved in the organization and regulation of partitioned tasks in distributed systems.

Seeley [24, 25] described in honey bees the “information-center strategy” as a concept of colony-level regulation. He showed that regulation of worker behavior depends upon a network of worker interactions that sets positive and negative feedbacks into the regulation mechanism. Karsai and Wenzel [26] showed that *Metapolybia* wasps use an information center to regulate their partitioned work for constructing the nest. *Metapolybia* and *Polybia* wasp societies are especially suitable to study mechanisms of task partitioning, because the workers exhibit neither morphological differences nor high degrees of temporal polyethism. Instead the workers exhibit adaptive and flexible task specialization in which distinct subsets of complex tasks such as nest construction are partitioned between cooperating teams of nest mates [27, 26, 28]. The information center in these wasps is the fill level of the common stomach [7, 26, 28, 29, 30, 31]. This common stomach emerges simply from the interaction of the workers and the need of temporal storage of water. The common stomach is a platform of worker connectivity [32] where pairs of individuals exchange water (direct interaction between a forager and the water storer wasp). The crops of some workers together comprise the “common stomach” or “social crop” of the colony where water is stored temporarily. We showed that the key role of the common stomach is to provide indirect interactions between the water providers and users, and this in turn provides a buffer against fluctuations and regulates the construction behavior [26, 30].

The construction of the nest requires 3 tasks and 2 materials. The nest builder (NB) wasps need wood pulp to work with. This wood pulp is provided by the pulp forager (PF) wasps, but for pulp foraging these wasps need water. The water is provided by the water forager (WF) wasps, but in an indirect manner. Water is not (or very rarely) transferred between water and pulp forager wasps. Instead WF wasps drop their water load into common stomach (CS) wasps, and PF wasps receive water from these individuals. The number of CS wasps is larger than the number of foragers, and the crops of these CS wasps together comprise the common stomach that stores water temporarily. A single interaction of the forager with a CS wasp can succeed (some water is transferred) or can fail (the CS wasp refuses to interact). In either case, the forager can continue to interact with other individuals or can abandon foraging. It seems these decisions depend on the numbers of successes and failures that in turn depend on the state of the common stomach [26]. This arrangement also ensures some delays and multiple interactions among the individuals, which is important to obtain a more reliable information acquisition in task partitioned systems of insect societies [33, 34].

Field studies and models showed that *Metapolybia* wasp colonies are able to adapt to internal and external perturbation, and using the common stomach as an information center, jobs that are in high demand will be filled and those in low demand are abandoned [26, 30, 31]. It was shown that the colony is a mix of workforces that ensures steady construction while keeping the dangerous foraging trips low and keeping the number of interactions between wasps higher than the minimum [29]. The goal of the current model we present here is different. Instead of focusing on modeling the task change itself, here we provide a detailed mathematical description of the possible mechanisms of the water exchange itself. Previous assumptions of our previous models are reevaluated, and with the use of mathematical reasoning and derivations, we will provide a more detailed account of the individual-level behavior and processes. Our specific hypotheses are the following: 1) Water exchange between foragers and the common stomach is modulated by a tradeoff between acceptance and resistance to water transfer; 2) The amount of water transferred in one interaction is dependent upon the status of the common stomach and the shape of the resistance function; 3) The numbers of successful and failed water transfers are dependent on the amount of water transferred in one interaction (that is, on the status of the common stomach and the shape of the resistance function); 4) Foraging can be aborted with high probability as the number of interactions becomes high. With this model we also seek answers to the following questions about colony-level phenomena: 1) For what reason in our previous models did the fill level of the common stomach stabilized just below 50%? 2) Why do pulp foragers need more interactions with the common stomach than do water foragers? 3) Why do foragers complete on average 5–7 interactions with the common stomach in natural colonies? 4) What explains the amount of variation in the number of successful interactions that foragers complete, and how might this be related to the fluctuating common stomach water level?

## The Model

### 1. Water Exchange between Individuals: The Resistance Function

#### 1.1. The common stomach: Symmetric buffering and general form of the resistance function

Let us define the water level *w* of a wasp to be the fraction of its crop volume that is filled with water. We assume that all wasps have the same maximum capacity for storing water. Consider a set of *N* CS wasps. We assume that all CS wasps have the same water level *w* so that the water level of the common stomach as a whole is also *w*.(Table 1)

**Table 1:** Definition of variables.

Name	Definition
*E*	percent difference in the values of *f* and $\bar{f}$ (function of *S*, *r*, *K*, and Δ*t*).
*F*_%_	failure rate of a forager; fraction of attempted interactions that failed (function of *s*).
*f*	number of failed interactions that a WF wasp makes in one foraging cycle, derived from empirical data (function of *s*).
$\bar{f}$	average number of failed interactions that a WF wasp makes in one foraging cycle, calculated under *P* (function of *s*).
*K*	probability that a forager will decide to attempt another interaction after a failure.
*n*	total number of attempted interactions in one foraging cycle (function of *s*).
*P*	probability distribution at a given water level over the number of successes *s* and number of failures *f* that a WF wasp will make.
*r*	probability that a CS wasp will refuse to interact with a WF wasp; probability that a WF wasp will make a failure (function of *w*).
*r*_*p*_	probability that a CS wasp will refuse to interact with a PF wasp; probability that a PF wasp will make a failure (function of *w*).
*S*	number of successful interactions needed for a WF wasp to completely rid its initial load of water (function of *w*).
*S*_*p*_	number of successful interactions needed for a PF wasp to completely fill its initially empty crop (function of *w*).
*s*	number of successful interactions that a WF wasp manages to complete in one foraging cycle before either ridding its entire load or prematurely aborting (function of *w*).
$\bar{s}$	average number of successful interactions that a WF wasp manages to complete in one foraging cycle before either ridding its entire load or prematurely aborting, calculated under *P* (function of *w*).
*s*_*p*_	number of successful interactions that a PF wasp manages to complete in one foraging cycle before either filling its entire crop or prematurely aborting (function of *w*).
*t*	time needed for a forager to complete one foraging cycle (function of *s*).
*w*	common stomach water level; fraction of the common stomach that is filled with water.
$\bar{w}$	average common stomach water level taken over time.
Δ*t*:	average time between two attempted interactions by a forager.
Δ*w*	fraction of one crop load of water that a WF wasp is able to transfer to an accepting CS wasp (function of *w*).
*σ*	curvature parameter for the function *r*.

A key function of the common stomach is to act as a buffer or regulator for the flux of water into and out of the colony via a negative feedback mechanism. If we introduce a WF wasp into the system, then the WF wasp selects and attempts to interact with a CS wasp in order to deposit water into the common stomach. Let *r*(*w*) be the probability that the CS wasp (with water level *w*) will resist or refuse to interact with the WF wasp. That is, *r*(*w*) is the probability that the WF wasp will "fail" when the common stomach is at a water level *w*. If the WF wasp fails to interact, then we call the attempt a "failure." Otherwise, the interaction is a "success." We call *r* the resistance function, and we assume that *r*(*w*) strictly increases as *w* increases; that is, a CS wasp with a greater water level is more likely to resist the in-flux of water. We also designate *r*(1) = 1, meaning that a full CS wasp is guaranteed not to accept water.

Suppose now that we introduce a PF wasp into the system. The PF wasp selects and attempts to interact with a CS wasp in order to withdraw water from the common stomach for its pulp-collecting trip. Since the common stomach is a buffering system, we assume that the ideal water level for this function is *w* = 0.5, which may or may not be the actual water level of the common stomach. If *w* = 0.5 is the ideal water level for buffering, then the CS wasp regards the WF wasp and PF wasp symmetrically; there is no bias towards one or the other. Thus, we assume that the situation of the PF wasp is identical to that of the WF wasp, except for the following: While the situation of the WF wasp depends on the water level *w*, the situation of the PF wasp, since it attempts to withdraw water, depends on the emptiness level 1 − *w*. Hence, by symmetry, resistance towards a WF wasp by a CS wasp with water level *w* is equivalent to the resistance to a PF wasp by a CS wasp with water level 1 − *w*; if *r*_*P*_ is the resistance towards a PF wasp, then *r*_*P*_(*w*) = *r*(1 − *w*).

A second key function of the CS wasp is to act as a mediator of water between the WF and PF wasps; the CS wasp accepts water from a WF wasp and then gives it to a PF wasp. We therefore assume in our model that a CS wasp with water level *w* is willing to interact with either a single WF wasp or a single PF wasp, and hence the CS wasp must resist either a WF wasp or a PF wasp but not both. That is, we exclude from our model the subset of CS wasps who refuse to interact. Furthermore, we assume that at any one time, a CS wasp is either willing to take more water or unload some water; that is, the CS wasp is willing to interact with either a WF wasp or a PF wasp, but not both. So, we have *r*(*w*) + *r*_*P*_(*w*) = 1. But since *r*_*p*_(*w*) = *r*(1 − *w*), then we have the following identity for the resistance function (Eq 1):

r(w)+r(1−w)=1,w∈[0,1] (1)

This identity involves the expected resistance towards a WF wasp as a function of the water level of the common stomach. Although we can replace *r* with *r*_*P*_ and water level with emptiness level for a PF wasp, we choose to focus only on the WF wasp. By symmetry, whatever results we obtain for WF wasps at a water level *w* = *x* can be transferred to PF wasps at the water level *w* = 1 − *x*.

By Eq 1, *r*(1) = 1 implies *r*(1 − 1) = *r*(0) = 0, meaning that an empty CS wasp is guaranteed to accept water and a full CS wasp is guaranteed to give water. Also, for *x* = 0.5, we have *r*(0.5) + *r*(1 − 0.5) = 1 so that 2*r*(0.5) = 1, implying *r*(0.5) = 0.5. This means that a CS wasp with *w* = 0.5 is equally likely to accept or give away water. More generally, Eq 1 implies the symmetry that a 180° rotation of the graph *y* = *r*(*x*) about the point (0.5, 0.5) leaves the graph unchanged. Therefore, *r* has the general form (Eq 2):

$$r(w)=\{\begin{array}{ll} g(w), & \text{if}\;0\leq w\leq 0.5\\ 1-g(1-w), & \text{if}\;0.5<w\leq 1 \end{array}\;,\;g(0)=0\;\text{and}\;g(0.5)=0.5$$ (2)

for some strictly increasing and differentiable function *g*. Note that *r* is differentiable at *w* = 0.5: Let $g^{\prime }(x)=\frac{\text{d}}{\text{d}w}g(w){|}_{w=x}$. So, we have $\frac{\text{d}}{\text{d}w}\left(1-g(1-w)\right){|}_{w=x}=g^{\prime }(1-x)$, and notice that *g*′(*x*) = *g*′(1 − *x*) for *x* = 0.5.

We assume that the shape of the graph *y* = *r*(*x*) is sigmoid since it captures both of the key functions of the CS wasp; the sigmoid shape is a strong regulator and tends to drive the system to a symmetric state (assuming no other asymmetric factors at this point) but still allows for enough fluctuation for the mediation of water. Thus, we choose the function *g* to be exponential, which has the general form *g*(*w*) = *a*⋅*b*^*w*^ + *c*. Now, since *g*(0) = 0, we have 0 = *a*⋅*b*^0^ + *c* = *a* + *c* so that *c* = −*a*. This gives *g*(*w*) = *a*⋅*b*^*w*^ − *a*. Now, since *g*(0.5) = 0.5, we have 0.5 = *a*⋅*b*^0.5^ − *a*, which results in $b={\left(1+\frac{1}{2a}\right)}^{2}$. Hence, we have:

$$g(w)=a{\left(1+\frac{1}{2a}\right)}^{2w}-a$$

where *a* is a free parameter. We find that as *a* increases, the steepness or sigmoidity of *y* = *r*(*x*) decreases. So, let *σ* = *a*^−1^ (parameter 1) be the curvature parameter, so that a greater value of *σ* implies greater steepness of the curve (Table 1). This gives (Eq 3):

$$g(w)=\sigma^{-1}{\left(1+0.5\sigma \right)}^{2w}-\sigma^{-1}$$ (3)

**Fig 1:**
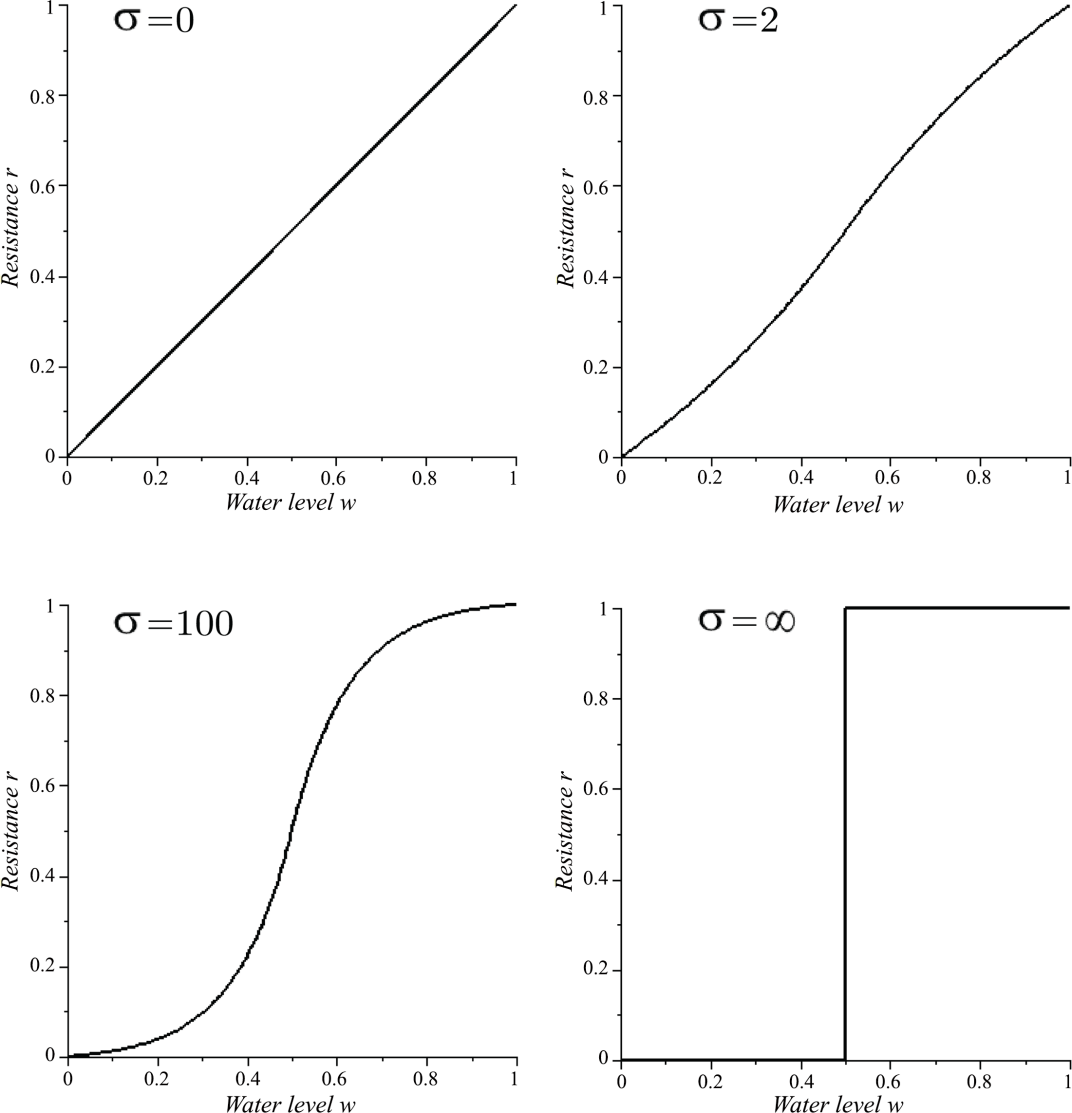
Resistance *r* as a function of the water level of the common stomach *w* and the curvature (steepness) parameter *σ* for the WF wasp (see Eqs 2–4). These functions describe the probability that a WF wasp fails in an interaction when the common stomach water level is *w* (under various values of *σ*).

Substituting this equation into Eq 2 then gives us the resistance function *r*. A greater water level *w* implies greater resistance and hence a greater probability that a WF wasp will fail to transfer water (Fig 1). As *σ* increases, the plot obtains a more "sigmoid" shape, which provides stronger water level regulation. The resistance function *r* for the PF wasp is *y* = *r*(1 − *w*); by symmetry, it is the reflection of the resistance function for a WF wasp about the line *w* = 0.5.

We also consider the limiting cases of the linear resistance function given by *r*_0_(*w*) = *w* (*σ* = 0) and the step resistance function given by (Eq 4):

$$r_{\infty }(w)=\{\begin{array}{ll} 0, & \text{if}\;0\leq w<0.5\\ 0.5, & \text{if}\;w=0.5\\ 1, & \text{if}\;0.5<w\leq 1 \end{array}\;(\sigma =\infty )$$ (4)

Thus, for *σ* = *∞*, a WF wasp is guaranteed to succeed for *w* < 0.5 and guaranteed to fail for *w* > 0.5.

#### 1.2. The quantity of water transferred between individuals depending on the water level and resistance

In this section, we derive how much water is transferred in one successful interaction between a forager and CS wasp as a function of common stomach water level. This will help to determine the number of successful interactions that a WF wasp must make to rid its water load. Suppose a CS wasp with water level *w* accepts water from a WF wasp. Let Δ*w* be the amount of water (expressed as a fraction of crop volume) transferred from the WF wasp to the CS wasp in their interaction, and let Δ*w* be a function of *w*. We assume that the CS wasp (and not the WF wasp) determines Δ*w*; we assume that a WF wasp will always try to empty its load in one interaction (Δ*w* = 1) (maximizing its task performance) but that the CS wasp is the one that ultimately decides how much water to accept from the WF wasp in accordance with its own internal state. As a second negative feedback mechanism to control water in-flux, we propose that Δ*w* strictly decreases as *w* increases. Again, since a full CS wasp accepts no water, define Δ*w*(1) = 0.

We construct a relationship between *w* and Δ*w* based on the following hypothetical scenario: Consider a hypothetical CS wasp that initially holds no water in its crop. Suppose the CS wasp successfully interacts with a series of WF wasps (one at a time) and accepts an average of Δ*w* units of water from each WF wasp. By the resistance function, the CS wasp will eventually resist further interaction with another WF wasp. By this point, the CS wasp has some final or "terminal" water level $\bar{x}$ (the terminal water level is an average because resistance is probabilistic). The terminal water level $\bar{x}$ is therefore a function of the increment Δ*w*. In particular, if we refer to the above scenario as "Scenario A", then we have that Scenario A "maps" the increment Δ*w* to the terminal water level $\bar{x}$. We will say that the increment Δ*w* "generates" the terminal water level $\bar{x}$.

Now consider a CS wasp with water level *w*. How much water will the CS wasp accept (assuming it does not resist)? That is, what is the value of Δ*w*(*w*)? The central hypothesis of our model is that the value of Δ*w*(*w*) is such that it "generates" the terminal water level $\bar{x}=1-w$. In other words, Scenario A "maps" the increment Δ*w*(*w*) to the terminal water level $\bar{x}=1-w$. More explicitly, we propose that a CS wasp with water level *w* accepts Δ*w*(*w*) units of water such that if a (different) hypothetical CS wasp is initially empty and accepts water in increments of Δ*w*(*w*) units, then it will ultimately have a terminal water level of $\bar{x}=1-w$.

Our central hypothesis is based on the fundamental symmetry between WF wasps and PF wasps. Since a CS wasp with water level *w* accepts Δ*w*(*w*) units of water from a WF wasp, then by symmetry a CS wasp with water level 1 − *w* transfers Δ*w*(*w*) units of water to a PF wasp. But by our central hypothesis, Δ*w*(*w*) "generates" the water level $\bar{x}=1-w$. Therefore, a CS wasp with water level 1 − *w* transfers Δ*w*(*w*) units of water to a PF wasp, where Δ*w*(*w*) is precisely the increment by which an empty hypothetical CS wasp must accept water from WF wasps in order to reach a terminal water level of $\bar{x}=1-w$.

The above scheme can be used to construct a mathematical relationship between *w* and Δ*w* as follows: Let $\frac{1}{\Delta w}\in Z^{+}$. Consider an initially empty CS wasp that has already accepted *i* − 1 water transfers of size Δ*w* from WF wasps. Thus, the CS wasp has water level *x*_*i*−1_ = (*i* − 1)Δ*w*. By definition of the resistance function, the probability that the CS wasp will accept an *i*^th^ transfer is 1 − *r*(*x*_*i*−1_) = *r*(1 − *x*_*i*−1_) = *r*(1−(*i* − 1)Δ*w*) (using Eq 1). Suppose the CS wasp continues to accept water until it has water level *x*. So, the CS wasp accepted $\frac{x}{\Delta w}$ transfers and refused the next transfer, the probability of which is *r*(*x*). To find the probability *P*_*w*_(Δ*w*, *x*) that an empty CS wasp accepts water in increments of size Δ*w* until it has water level *x*, we multiply the probabilities of the CS wasp accepting the first $\frac{x}{\Delta w}$ transfers and resisting the next transfer:

$$P_{w}(\Delta w,x)=r(x)\prod_{i=1}^{\frac{x}{\Delta w}}r\left(1-(i-1)\Delta w\right)$$

This is a probability density function over *x* and therefore sums to 1 over *x* for a given Δ*w*. It is similar to the geometric distribution but differs in that 1) there is an upper limit to the number of successes (once the CS wasp is full) and 2) the probability of success changes in accordance with the continuous resistance function. Every possible water level can be written as *j*Δ*w*, $j\in Z^{+}\;:1\leq j\leq \frac{1}{\Delta w}$. Notice that *j* = 0 is not possible since an empty CS wasp must accept the first transfer due to 1 − *r*(0) = 1. So, the average water level $\bar{x}$ at which the in-flux of water into the CS wasp terminates is:

$$\bar{x}(\Delta w)=\sum_{j=1}^{\frac{1}{\Delta w}}P_{w}(\Delta w,j\Delta w)\cdot j\Delta w$$

According to our central hypothesis, a CS wasp with water level *w* accepts an amount Δ*w* of water such that the terminal water level of an initially empty CS wasp accepting water in increments of Δ*w* is $\bar{x}=1-w$, and hence $w=1-\bar{x}$. Thus, we have the following relationship between Δ*w* and *w* (Eq 5):

$$w(\Delta w)=1-\sum_{j=1}^{\frac{1}{\Delta w}}P_{w}(\Delta w,j\Delta w)\cdot j\Delta w$$ (5)

Although we have *w* as a function of Δ*w*, it is difficult to derive a closed-form expression for the inverse function that gives Δ*w* in terms of *w*. So, to evaluate Δ*w*(*x*), we adjust *x* until *w*(Δ*w*) = *x*.

The equation Eq 5 reduces and simplifies in the special case of *r*_∞_. This is significant because this limiting case defines a lower bound on Δ*w*(*w*), which can be used to place a lower bound on the average water level $\bar{w}$ of the common stomach. Let $\Delta w=\frac{1}{n},n\in Z^{+}$. Suppose *n* is even. So, $n=2k,k\in Z^{+}$. This means $\Delta w=\frac{1}{2k}$ so that *k*Δ*w* = 0.5. Thus, 0.5 is an integral multiple of Δ*w*.

Since *r*_∞_(*x*) = 0 for *x* ∈ [0, 0.5), a CS wasp is guaranteed to reach a water level *x* = 0.5 if it accepts water in increments of Δ*w*. Now, since *r*_∞_(0.5) = 0.5, there is a 50% chance that the CS wasp will accept another Δ*w*. If it does not accept, then its final water level is *x* = 0.5. If it accepts, then its final water level is *x* = 0.5 + Δ*w*, after which it can accept no more since *r*_∞_(*x*) = 1 for *x* ∈ (0.5, 1]. Thus, the average water level is $\bar{x}=0.5(1+\Delta w)$. Since $w=1-\bar{x}$, we have *w* = 1 − 0.5(1 + Δ*w*) = 0.5(1 − Δ*w*).

Suppose *n* is odd. So, $n=2k-1,k\in Z^{+}$. This means $\Delta =\frac{1}{2k-1}$ so that 2*k*Δ*w* − Δ*w* = 1 or *k*Δ*w* = 0.5(1 + Δ*w*). Since *r*_∞_(*x*) = 0 for *x* ∈ [0, 0.5), the CS wasp is guaranteed to reach a water level *x* = 0.5(1 + Δ*w*). Since 0.5(1 + Δ*w*) > 0.5, *r*_∞_(0.5(1 + Δ*w*)) = 1 so that this is the final water level $\bar{x}$. So, $w=1-\bar{x}=1-0.5(1+\Delta w)=0.5(1-\Delta w)$. Therefore, we have for all $n\in Z^{+}$ the following relationship (Eq 6):

$$w(\Delta w)=\frac{1-\Delta w}{2}\;\text{and}\;\Delta w(w)=1-2w,\;w\in [0,0.5]$$ (6)

Notice that Δ*w*(0.5) = 0 so that a CS wasp with water level *w* = 0.5 neither accepts nor gives water. Thus, if the common stomach is 50% full, then all interactions stop, assuming no asymmetric factors exist (of course, this is only in the limiting case).

**Fig 2:**
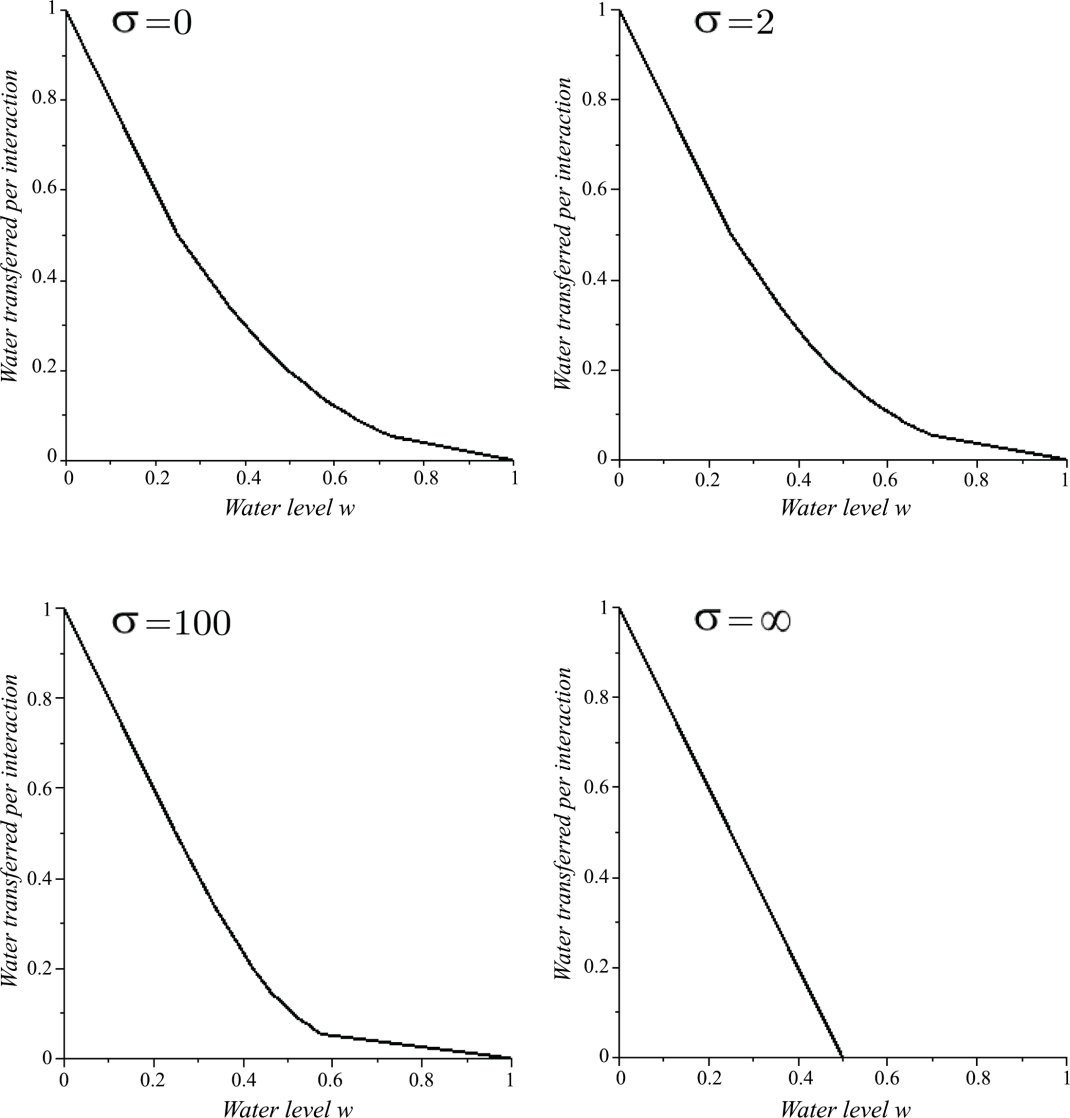
The amount of water Δ*w* transferred in a successful interaction by a WF wasp as a function of the water level of the common stomach *w* and the curvature (steepness) parameter *σ* (see Eqs 5 and 6).

The amount of water Δ*w*(*w*) transferred in one successful interaction decreases with the water level *w* (Fig 2). As *σ* increases, Δ*w*(*w*) decreases for all 0.25 < *w* < 1; stronger resistance is accompanied by less water acceptance. Note that the plots for *σ* = 0 and *σ* = *∞* are natural upper and lower bounds on Δ*w*(*w*) respectively. A similar function for a PF wasp on receiving water from a CS wasp can be obtained by reflecting *y* = Δ*w*(*w*) about *w* = 0.5.

Notice that Δ*w*(0) = 1 for all *σ*, which means that an empty CS wasp accepts all the water from a WF wasp, and a full CS wasp gives all of its water to a PF wasp. Thus, it is possible for an empty CS wasp to simply alternate between *w* = 0 and *w* = 1, although other factors break this alternation. Also, note that Δ*w*(0.25) = 0.5 for all *σ*; when the common stomach is one-quarter full, a WF wasp will be able to transfer half of one crop load of water to an accepting CS wasp regardless of resistance (Fig 2). This can be seen as follows: Consider an empty CS wasp. Suppose it accepts water in increments of Δ*w* = 0.5. Since the CS wasp is initially empty, then it is guaranteed to accept the first transfer. Since *r*(0.5) = 0.5 for all *σ*, there is a 50% chance that the CS wasp will accept a second water transfer. Thus, there is a 50% chance that the CS wasp will remain at *x* = 0.5 and a 50% chance that it will rise to *x* = 1. Thus, on average, we have $\bar{x}=0.75$ so that $w=1-\bar{x}=0.25$.

### 2. Multiple Interactions of a Foraging Wasp with Common Stomach Wasps: Successes and Failures

#### 2.1. The number of successes needed for a water forager to deliver its full load

We showed that if the common stomach has water level *w*, then a WF wasp is able to deposit Δ*w*(*w*) of its water into the common stomach per successful interaction with a CS wasp (see Eq 5). Thus, for a WF wasp to completely empty itself in one cycle of interactions, the number of successful interactions that it must make is given by (Eq 7):

$$S(w)=\text{ceil}\left(\frac{1}{\Delta w(w)}\right),$$ (7)

where "ceil" denotes the ceiling function. The ceiling function is used because a WF wasp can only make an integral number of interactions, even if a bit of water remains for the last interaction. Here, we define one foraging cycle to be the set of interactions attempted for one cropload of water; it is the period between foraging trips. Again, the CS wasps force a WF wasp to make *S* successful interactions via resistance; since the CS wasps determine Δ*w*, they determine *S*.

In the special case of *σ* = *∞*, the number of successes *S*_∞_ needed for a WF wasp to empty itself is given by (Eq 8):

$$S_{\infty }(w)=\text{ceil}\left(\frac{1}{\Delta w(w)}\right)=\text{ceil}\left(\frac{1}{1-2w}\right),$$ (8)

where Δ*w* is given by Eq 6.

**Fig 3:**
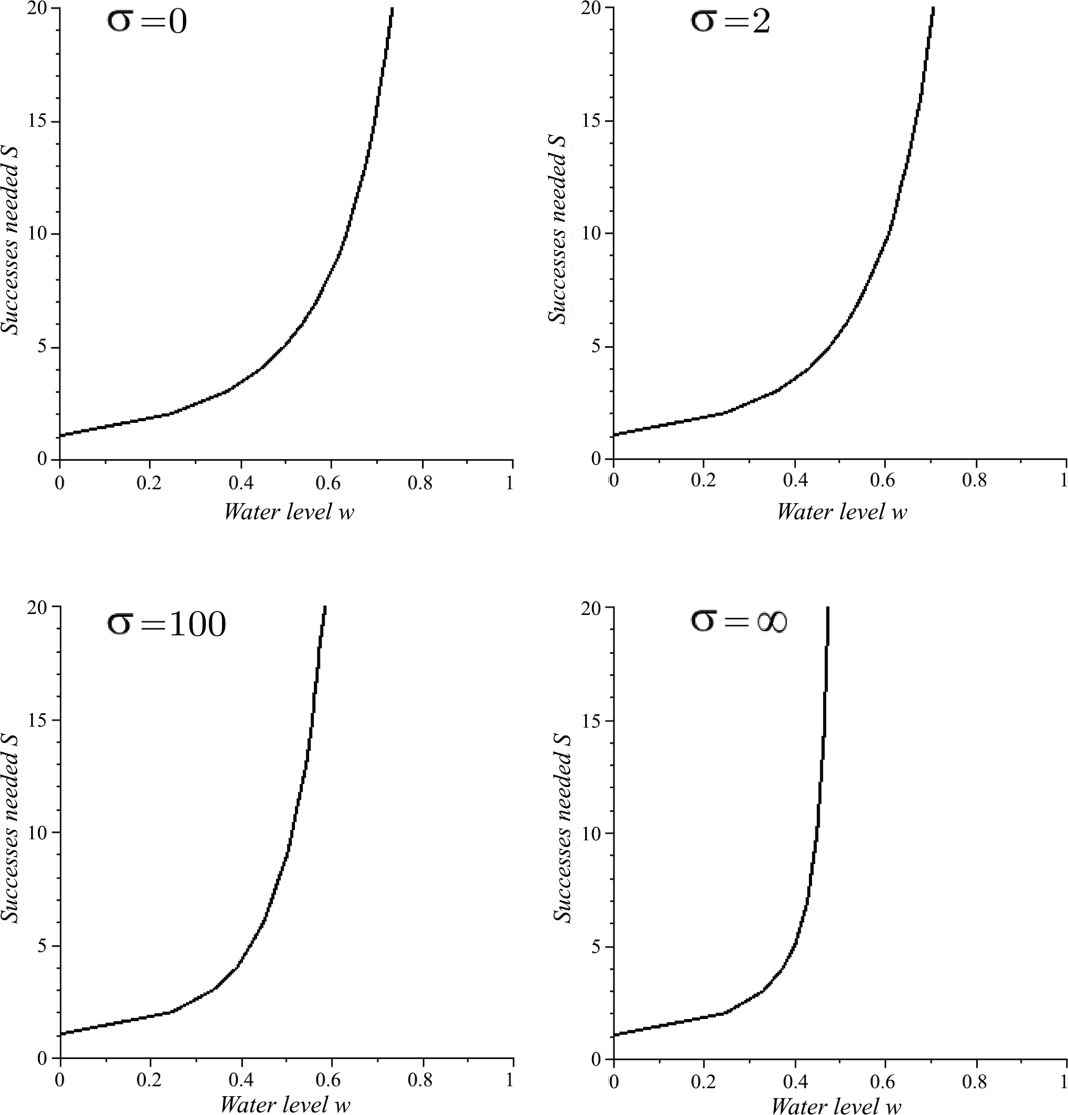
The number of successes *S* needed for a WF wasp to completely unload its full crop content while interacting with CS wasps as a function of the water level of the common stomach *w* and the curvature (steepness) parameter *σ* (Eqs 7 and 8). For clarity, the effect of the ceiling function (used in Eqs 7 and 8) is not shown.

As the water level of the common stomach *w* increases, the number of successes needed *S* increases quickly (Fig 3). For clarity, the effect of the ceiling function (included in the definition of *S*) is not shown in Fig 3. A greater water level implies a greater number of successes needed for complete unloading since CS wasps accept less water at greater water levels. The plot is undefined when the common stomach is full since no successes can be made. As *σ* increases, *S*(*w*) increases for all 0.25 < *w* < 1; under stronger resistance, more successes are needed for complete unloading. Note that *S*(0.25) = 2 for all *σ*; when the common stomach is one-quarter full, 2 successes are needed for complete unloading. The plots for *σ* = 0 and *σ* = *∞* are natural lower and upper bounds respectively on *S*(*w*). For *σ* = *∞*, *S*(*w*) is undefined for *w* ≥ 0.5 because Δ*w*(0.5) = 0 (under *σ* = *∞*) and successes are impossible for *w* > 0.5 due to resistance under *σ* = *∞*.

A similar function for the number of successes needed *S*_*P*_ for a PF wasp to completely fill its empty crop can be obtained by reflecting *y* = *S*(*w*) about *w* = 0.5 since *S*_*P*_(*w*) = *S*(1 − *w*) (where *S* was defined in Eq 7).

Until a WF wasp makes *S* successes, it attempts a number of interactions per cycle, some of which succeed and others fail due to resistance from the CS wasp. Let *s* be the number of successes that a WF wasp makes in one foraging cycle, where *s* is a function of the water level *w* of the common stomach (Table 1). It is not necessary that *s* = *S*, as we will see. Let *f* be a function such that *f*(*s*) is the expected number of failures that a WF wasp will make if it makes *s* successes. So, the total number of interactions per cycle that a WF wasp makes is *n*(*s*) = *s* + *f*(*s*). Now, we can define the failure rate (percentage of interactions that fail) for a WF wasp as follows:

$$f_{\%}(s)=\frac{f(s)}{n(s)}$$

In all of the above, we can replace *s* with *s*_*P*_ in the case of a PF wasp.

It is possible for a WF wasp to interact with the same CS wasp more than once per foraging cycle (although not consecutively). Thus, in the theory so far, it is possible for a wasp to fail every interaction that it attempts, so that it never completes the *S* successes needed for emptying and is trapped in the same foraging cycle forever; *n* = *∞*. To avoid such infinities, we assume that there exists a function, call it cont, such that cont(*s*, *f*) is the probability that a WF wasp will attempt another interaction, having already made *s* successes and *f* failures. So, if the total number of interactions is high, then it is possible for a WF wasp to abort its cycle of interactions before it completes *S* successes. Thus, *s*(*w*) ≤ *S*(*w*). Note that in nature, a WF wasp that carries unloaded water and aborts the water foraging task tends to switch tasks, e.g., to a CS wasp, but our model does not address this behavior. We have investigated this in detail as reported previously [26, 30, 31, 35]. An explicit form of the cont function is presented next.

#### 2.2. Probability that a foraging wasp prematurely aborts its foraging cycle: The cont function

Let cont be a function such that cont(*s*, *f*) is the probability that a wasp that has completed *s* successes and *f* failures will continue and seek out another CS wasp with which to interact. It is important that this function not be strictly 1, since it is otherwise possible for a wasp to continuously make failures such that it never completes its *S* necessary successes.

We know that if *s* = *S*, then the wasp is done with its foraging cycle and does not look for another CS wasp. Thus, cont(*S*, *f*) = 0. Let *K* (parameter 2) be the probability that a wasp will decide to attempt another interaction after a failure (after being rejected by a CS wasp). Thus, the probability that a wasp will decide to attempt another interaction after making *f* failures is *K*^*f*^. Therefore, the probability that a wasp will attempt another interaction after having completed *s* successes and *f* failures is given by the following (Eq 9):

$$\text{cont}(s,f)=\{\begin{array}{ll} 0, & \text{if}\;s=S\\ 1, & \text{if}\;f\leq 0\\ K^{f}, & \text{if}\;s<S\;\text{and}\;f>0 \end{array}$$ (9)

Notice that 1 − cont is a probability mass function. We defined cont for *f* ≤ 0 so that we can later take the delta of cont to obtain a probability density function.

We assume that the value of *K* is constant and is independent of the common stomach water level; every time a forager fails to interact, it "rolls a die" to decide whether it should attempt another interaction or abort. In real systems, for any number of observed failures (by any set of foragers), *K* is the fraction of the failures that were followed by the decision to continue. Note that since *K* is independent of the water level, we do not have to assume that foragers "read" the common stomach water level. This is important because, for example, there can exist cases when a forager makes no successes, even if the common stomach has the capacity to receive water (since resistance is probabilistic), but this forager must still nevertheless decide whether to continue or abort its current interacting behavior.

### 3. Constructing a Probability Distribution overall Success-Failure Combinations

Given a particular water level *w*, we now define the function *P* such that *P*(*s*, *f*) is the probability that a WF wasp will make *s* successes and *f* failures in its foraging cycle. The same probability holds for a PF wasp at a water level 1 − *w*. The construction of *P* incorporates the resistance function (*r*), the number of successes needed for emptying (*S*), and the probability that a wasp attempts another interaction (cont(*s*, *f*)). The distribution function *P* is a complete theoretical description of foragers and their interactions with CS wasps. We will use *P* to calculate quantities such as the average number of successes and average number of failures that a wasp makes at a given water level. These calculated quantities will later be used to study the effects of our parameters on the model and to compare the model predictions with empirical data.

Let *R* = *r*(*w*), with *w* a fixed water level. The probability that a WF wasp makes *s* successes is (1 − *R*)^*s*^, and the probability that it makes *f* failures is *R*^*f*^. The number of ways that a WF wasp can make *s* successes and *f* failures is $\left(\begin{array}{c} s+f\\ s \end{array}\right)$. Based on these probabilities alone, the probability that a WF wasp will make *s* successes and *f* failures is $\left(\begin{array}{c} s+f\\ s \end{array}\right){\left(1-R\right)}^{s}\;R^{f}$.

We must modify the above probability result to incorporate the number of successes *S* needed for emptying. The necessary modification is understood by examining the interaction that concludes the foraging cycle of the WF wasp: If *s* = *S*, then the concluding interaction was necessarily a success, the probability of which is $\frac{s}{s+f}$. In addition, a wasp with *s* = *S* continued after *s* − 1 successes and *f* failures but not after *s* successes and *f* failures. The probability of this is cont(*s* − 1, *f*) − cont(*s*, *f*). In contrast, *s* < *S* means that the last interaction was a failure, the probability of which is $\frac{f}{s+f}$. Also, a wasp with *s* < *S* continued after *s* successes and *f* − 1 failures but not after *s* successes and *f* failures. The probability of this is cont(*s*, *f* − 1) − cont(*s*, *f*). We can collect these different probabilities into a function called last that describes the concluding interaction as follows:

$$\text{last}(s,f)=\{\begin{array}{ll} 0, & \text{if}\;s+f=0\\ \left(\frac{s}{s+f}\right)\left(\text{cont}(s-1,f)-\text{cont}(s,f)\right), & \text{if}\;s=S\\ \left(\frac{f}{s+f}\right)\left(\text{cont}(s,f-1)-\text{cont}(s,f)\right), & \text{if}\;s<S \end{array}$$

Notice that we also defined the probability to be 0 if *s* + *f* = 0 to eliminate wasps who attempt no interactions.

Combining the above probabilities, the probability that a WF wasp will make *s* successes and *f* failures is proportional to the function:

$$p(s,f)=\left(\begin{array}{c} s+f\\ s \end{array}\right){\left(1-R\right)}^{s}\;R^{f}\;\text{last}(s,f)$$

To obtain the desired probability distribution *P*, We must normalize this function, which requires us to sum *p*(*s*, *f*) over all (*s*, *f*). The limits on the summation are 0 ≤ *s* ≤ *S* and 0 ≤ *f* < *∞*. Because of the cont function, we know that $\underset{f\to \infty }{\text{lim}}\;p(s,f)=0$. Thus, to reduce computation time, we can define a finite upper limit max(*f*) on *f* such that *p*(*s*, max(*f*)) ≈ *p*(*s*, *∞*). Thus, 0 ≤ *f* ≤ max(*f*). We will use max(*f*) = 200, since we can be almost certain that no wasp will continue after 200 failures [26]. The normalization constant (*A*) is therefore:

$$A=\sum_{j=0}^{\text{max}(f)}\sum_{i=0}^{S}p(i,j)$$

With this, the probability that a WF wasp at a water level *w* will make *s* successes and *f* failures in its foraging cycle is given by (Eq 10):

$$P(s,f)=\frac{p(s,f)}{A}$$ (10)

#### 3.1. The average number of successes and average number of failures

The average number of successful interactions $\bar{s}$ completed by a WF wasp at a water level *w* is given by (Eq 11):

$$\bar{s}(w)=\sum_{j=0}^{\text{max}(f)}\sum_{i=0}^{S}P(i,j)\cdot i$$ (11)

where we make it explicit that $\bar{s}$ is a function of the water level *w* because *R* = *r*(*w*) and *S* are functions of *w*.

The average number of failed interactions $\bar{f}$ experienced by a WF wasp at a water level *w* is given by (Eq 12):

$$\bar{f}(w)=\sum_{j=0}^{\text{max}(f)}\sum_{i=0}^{S}P(i,j)\cdot j$$ (12)

where again $\bar{f}$ is a function of the water level *w*.

The above functions (including the distribution *P*) depend on the resistance *r*(*w*) (and therefore ultimately on the parameter *σ*) and the parameter *K*. To find the value of (*σ*, *K*) that correctly describes real systems, we examine empirical data from real systems and present derivations based on them in the following sections.

### 4. Number of Successful Interactions as a Function of the Common Stomach Water Level

In Section 1.1, we proposed that the water level determines the probability that a CS wasp will resist interaction and cause the attempted interaction to fail. In Section 1.2, we proposed that the water level also determines the amount of water that a CS wasp will accept/give, thereby determining the number of successes needed for a foraging wasp to complete its cycle. Together, these two propositions imply that the water level determines the number of interactions (successes + failures) needed for a wasp to complete its foraging cycle. We then proposed a programmed function of behavior (the cont function) that determines what fraction of these interactions a wasp is likely to complete before it decides to abort its cycle. Thus, in short, the common stomach determines the number of interactions needed, and the foraging wasp decides what fraction of these it will complete before aborting. Hence, the value of *s* is an indicator of the water level of the common stomach.

**Fig 4:**
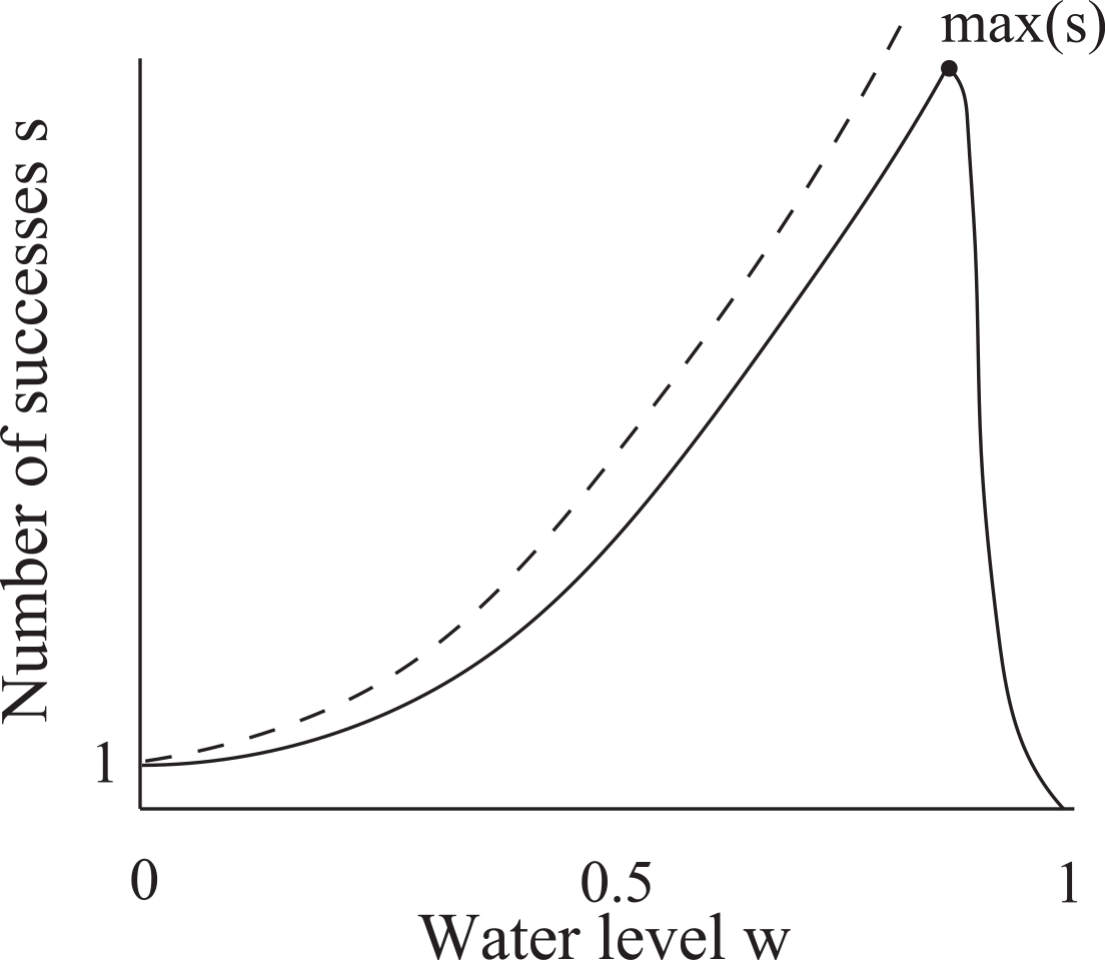
Schematic sketch of the number of successes *s* that a WF wasp is expected to complete when the common stomach water level is *w* (solid curve). An upper bound to *s* is the number of successes *S* that a WF wasp needs to completely rid its load (dashed curve). There exists a water level at which a WF wasp is expected to make a maximum number of successes max(*s*). The plot for the number of successes *s*_*p*_ expected for a PF wasp at common stomach water level *w* is obtained by *s*_*p*_(*w*) = *s*(1 − *w*). These curves are meant to convey only a general trend (described in Section 2.1) and are not plots of equations.

In this section, we place certain constraints on the water level *w* to help us with interpreting and using empirical data presented in later sections. The general behavior described in Section 2.1 allows us to draw a schematic sketch (i.e., the general trend) of the expected shape of the curve for *y* = *s*(*w*), where *w* is the water level of the common stomach (Fig 4). At *w* = 0, a WF wasp is guaranteed to succeed (no failures) so that *s*(0) = *S*(0) = 1. As *w* increases, *S* increases. To complete the larger number of successes *S* needed, a WF wasp must make more successes so that *s* increases. However, since *r* and therefore the failure rate *f*_%_ increase as *w* increases, it becomes more probable for a WF wasp to abort its cycle before completing *S* successes. Thus, *s*(*w*) < *S*(*w*) for *w* > 0. Eventually, *f*_%_ is so large that the WF wasp begins to abort its cycle with fewer and fewer successes, so that there exists a water level at which *s*(*w*) has its maximum value max(*s*) and after which *s*(*w*) decreases. At *w* = 1, failure is guaranteed so that *s* = 0 (Fig 4). By symmetry, the number of successes *s*_*P*_ that a PF wasp is expected to make at a water level *w* is given by *s*_*P*_(*w*) = *s*(1 − *w*).

**Fig 5:**
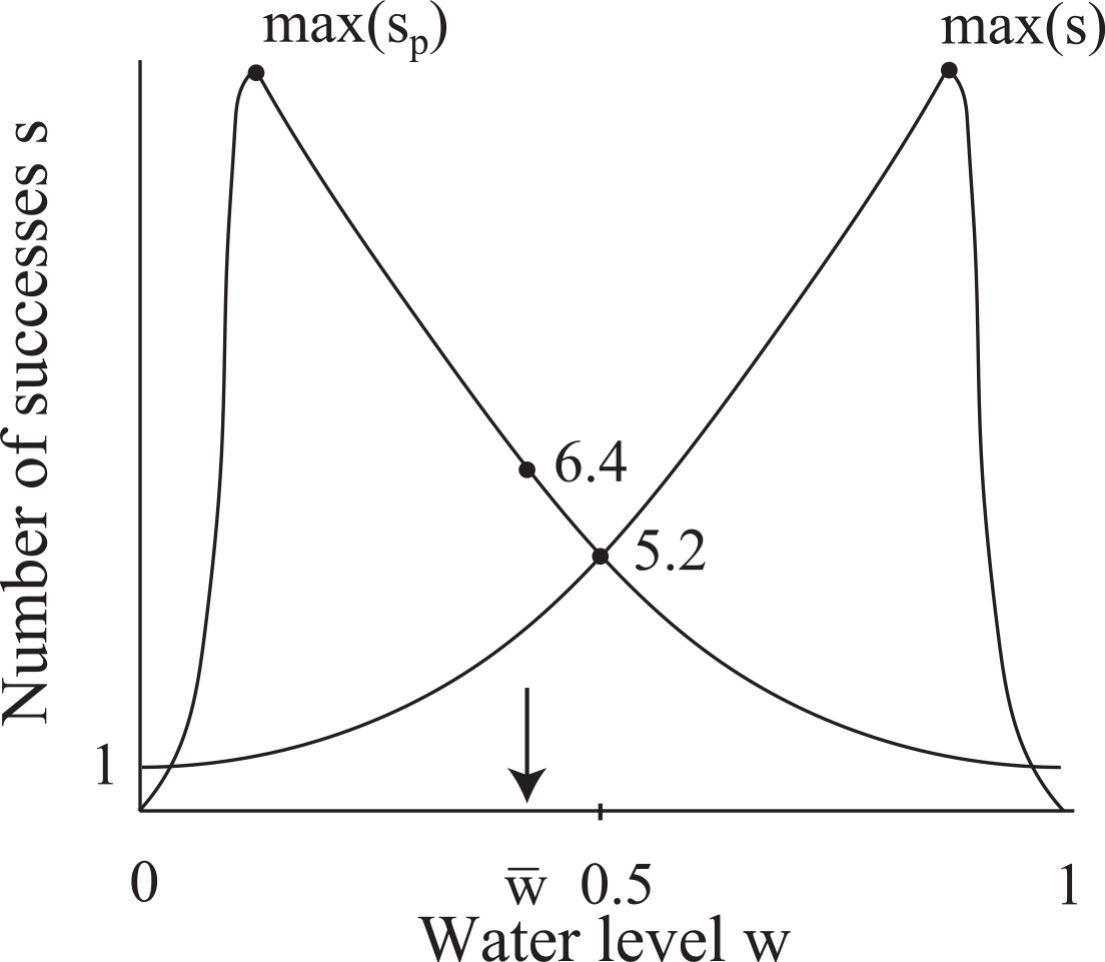
Schematic sketches for the number of successes *s* and *s*_*p*_ expected for a WF wasp and PF wasp to complete in one foraging cycle respectively when the common stomach water level is *w*. The established average water level of the common stomach is $\bar{w}$ (arrow), and it is assumed to have a value greater than where max(*s*_*p*_) occurs and has a value less than *w* = 0.5. The empirical average numbers of successes [26] for a WF wasp and PF wasp (5.2 and 6.4 respectively) occur at water levels greater and less than the average $\bar{w}$ respectively (this is deduced from the hypothesized shapes of the curves). Note that *s* = 5.2 might occur below, at, or above *w* = 0.5. Also, *s*_*p*_ = 6.4 occurs at a water level nearer to the average $\bar{w}$ than does *s* = 5.2. Note that $\bar{w}$ is located under a steeper section of *y* = *s*_*p*_(*w*) than of *y* = *s*(*w*), so that the deviation in *s*_*p*_ is greater than the deviation in *s* (4.3 > 2.8) in agreement with empirical findings of Karsai and Wenzel [26].

The schematic sketches of *y* = *s*(*w*) and *y* = *s*_*P*_(*w*) show that these functions are symmetric about *w* = 0.5 so that their absolute maxima occur at water levels equidistant from *w* = 0.5 (Fig 5). Let *w*(*x*) be the water level such that *s*(*w*(*x*)) = *x*. Since *s*_*P*_(*w*) = *s*(1 − *w*), 1 − *w*(*x*) is the water level such that *s*_*P*_(1 − *w*(*x*)) = *s*(*w*(*x*)) = *x*. Let $\bar{w}$ be the average water level of the common stomach taken over time. We assume that the average water level lies between the points at which the maximum numbers of successes occur: $1-w(\text{max}(s))<\bar{w}<w(\text{max}(s))$ (Fig 5). We assume that the water level fluctuates symmetrically about $\bar{w}$. We also assume that the feedback mechanisms that regulate the water level are robust and timely such that the water level never fluctuates beyond the points at which max(*s*_*P*_) and max(*s*) occur. Given that the common stomach is large and water flux is small in the sense that one foraging wasp is observed to make multiple interactions to withdraw/deposit at most one crop load of water, we are confident that our statement on the fluctuation of the water level is a safe assumption. It has been observed that the average numbers of successes (taken under fluctuating water level of the common stomach) that WF wasps and PF wasps make are 5.2 and 6.4 respectively [26]. Since 6.4 > 5.2, $\bar{w}$ must be such that it is more frequent to find *s*_*p*_(*w*) > *s*(*w*) than *s*_*p*_(*w*) < *s*(*w*). The symmetric fluctuation of water about $\bar{w}$ then implies $\bar{w}<0.5$ (Fig 5). For *w* < *w*(max(*s*)), we assume that the graph *y* = *s*(*w*) is primarily concave-up. This implies that the average number of successes for WF wasps occurs at a water level greater than the average water level: $w(5.2)>\bar{w}$. By symmetry, we have $1-w(6.4)<\bar{w}$. Thus, $1-w(6.4)<\bar{w}<w(5.2)$. Since the graph *y* = *s*(*w*) has a turning point at its maximum, its concavity must decrease to 0 at some point. Thus, the plot of *s*(*w*) is more linear at *w*(6.4) than at *w*(5.2). By symmetry, *s*_*p*_ is more linear at 1 − *w*(6.4) than is *s* at *w*(5.2). This implies that $\bar{w}$ is closer to 1 − *w*(6.4) than to *w*(5.2): $\bar{w}-(1-w(6.4))<w(5.2)-\bar{w}$. Solving for $\bar{w}$ from this inequality, we get (Eq 13):

$$\bar{w}<\frac{\left(1-w(6.4))-w(5.2\right)}{2}=\frac{1-(w(6.4)-w(5.2))}{2}$$ (13)

Note that since *w*(6.4) > *w*(5.2), *w*(6.4) − *w*(5.2) > 0 so that $\bar{w}<0.5$.

We want to derive an upper bound on *s*(0.5), which will later allow us to derive bounds on model parameters. Suppose 0.5 ≤ *w*(5.2). Since *s* is increasing, *s*(0.5) ≤ *s*(*w*(5.2)) = 5.2 < 5.8. Thus, *s*(0.5) < 5.8. Suppose, on the other hand, *w*(5.2) < 0.5. Since $1-w(6.4)<\bar{w}<w(5.2)$, we have 1 < *w*(5.2) + *w*(6.4), or 0.5 < 0.5(*w*(5.2) + *w*(6.4)). Let *L* be the secant line joining the points (*w*(5.2), 5.2) and (*w*(6.4), 6.4). Since *L* is increasing, *L*(0.5) < *L*(0.5(*w*(5.2) + *w*(6.4))). Since *L* is linear, *L*(0.5) < 0.5(*L*(*w*(5.2)) + *L*(*w*(6.4))) = 0.5(5.2 + 6.4). Thus, *L*(0.5) < 5.8. Since we assumed *s* to be concave-up, *s*(0.5) < *L*(0.5) so that *s*(0.5) < 5.8.

#### 4.1. The leaky common stomach

In Section 4, we showed that since the observed average number of successful interactions is smaller for WF wasps than for PF wasps (5.2 < 6.4), $\bar{w}<0.5$. This seems to contradict the previously assumed symmetry (Section 1.1) that the resistance function drives the common stomach to a water level of 0.5. To account for this, we implement the observation that the common stomach is "leaky"; that is, CS wasps not only store water but also consume it for purposes of drinking, cooling, etc. [26]. Since water cannot spontaneously appear in the common stomach, there is no counterbalance to the consumption of water (note that the water delivered by WF wasps is counterbalanced by the withdrawal of water by PF wasps). This means that this water leakage is an asymmetric property of the common stomach. Thus, since the resistance function tends to drive the common stomach to a water level of 0.5, the leak implies that the actual water level towards which the common stomach is driven is some value slightly less than 0.5.

If we assume that the water level fluctuates about $\bar{w}$ symmetrically, then the fact that $\bar{w}<0.5$ implies that the water level fluctuates under a steeper portion of *y* = *s*_*P*_(*w*) than of *y* = *s*(*w*) since these two functions are symmetric about *w* = 0.5 (Fig 5). Thus, the standard deviation in *s*_*P*_ ought to be greater than that in *s*, and indeed observations show that this is the case in natural colonies (average values of *s* and *s*_*p*_ taken under fluctuating water level are 5.2 ± 2.8 and 6.4 ± 4.3 respectively, 4.3 > 2.8) [26].

### 5. Correlation between Number of Successes and Interaction Time

**Fig 6:**
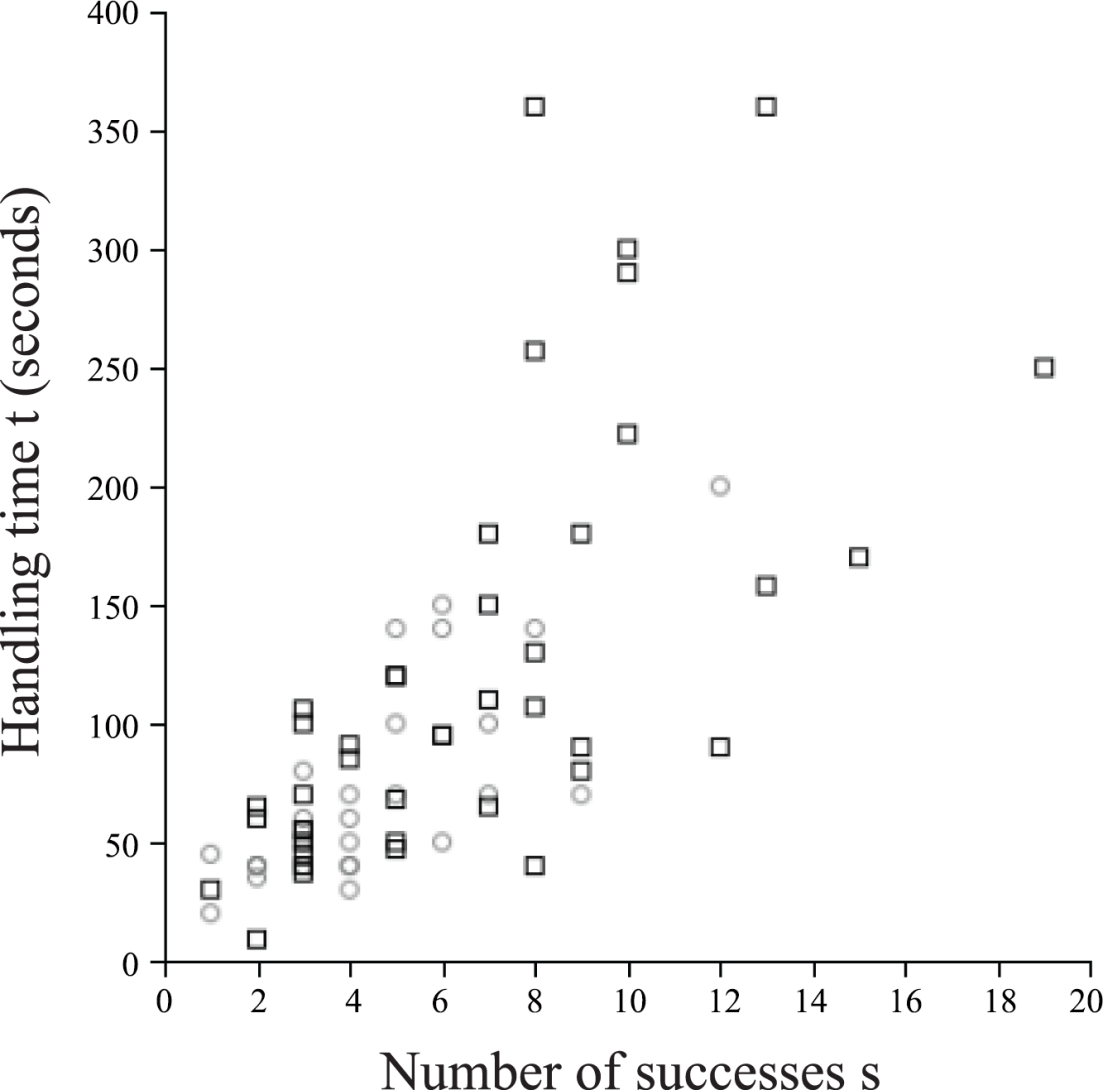
Empirical relationship [26] between the handling time *t* —i.e., total time needed for downloading (WF: grey circles) or uploading (PF: black squares) water in a single foraging cycle—and number of successes *s* completed during this behavior.

To obtain a correlation between the number of successes and the number of failures that a forager makes in one cycle, we used data from Karsai and Wenzel [26], where the number of successes *s* was measured (S1 File). In this section, we fit a function to this collected data set (*s*, *t*), where *s* is the number of successful interactions that a foraging wasp was observed to make in one cycle and *t* is the time (in seconds) that it took for the wasp to complete its cycle (Fig 6). We will use this function to construct a failure rate function, which will carry empirical information. This will be the function against which we can compare theoretical results. By the symmetry of WF wasps and PF wasps, the relationship between *s* and *t* ought to be the same for both WF wasps and PF wasps; the asymmetry due to the "water leakage" should only result in the PF wasps having a higher average value of *s*. This allows us to group the data for WF wasps and PF wasps together.

Overall, handling time increases with number of successes (Fig 6). If the data is taken to suggest that the rate of increase increases with number of successes, then the source of the increasing rate must be an increasing number of failures with successes (assuming average time between any two interactions is fixed). In particular, number of failures increases at an increasing rate with number of successes. Since resistance increases with water level, this suggests that number of successes increases with water level up to the water level where the maximum number of successes occurs. Thus, this data provides empirical support for some of our hypotheses (Section 1.2).

**Fig 7:**
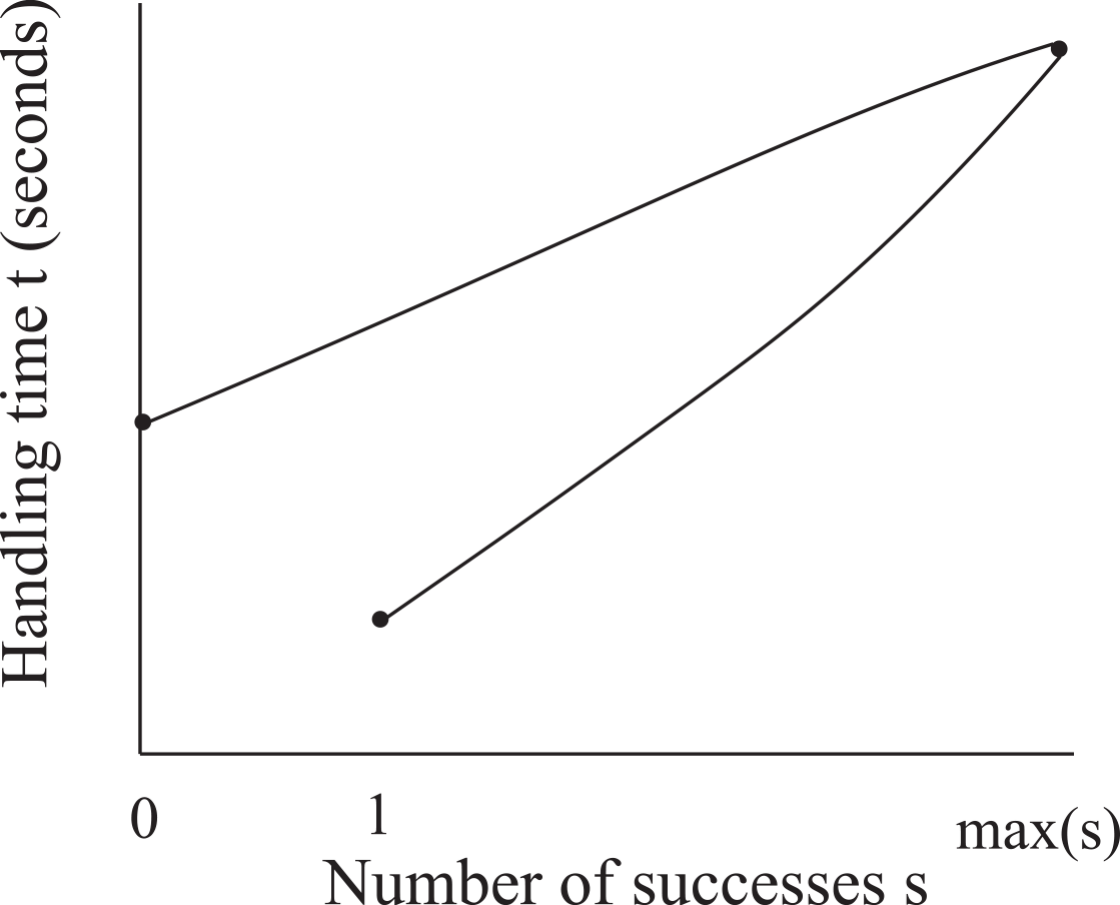
Schematic sketch of a possible fit to the handling time *t* vs. number of successes *s* data shown in Fig 6 assuming that the common stomach water level *w* fluctuates on both sides of the water level at which the maximum number of successes is made (see Fig 4). If the water level could fluctuate beyond the point where max(*s*) occurs, then two distinct water levels could have the same number of successes. These distinct water levels correspond to different handling times *t* since the number of failures varies with water level. Thus, for 1 ≤ *s* < *S*, one value of *s* corresponds to two values of *t* (as shown). The double curve depicted is ultimately not used for fitting, since we assume the water level never fluctuates to the extent described above (Section 4).

We now want to fit a curve to the data and obtain *t* as a function of *s*. In Section 4, we show that for 1 ≤ *s* < max(*s*), there are two water levels at which a given value of *s* occurs (Fig 5). Since there are two different resistances at these two water levels, we find that for one value of *s*, there are two distinct values of *n*(*s*) (total number of interactions). If we assume that the time required to interact with one CS wasp is a constant Δ*t*, then *t* and *n* are linearly related. Thus, for 1 ≤ *s* < max(*s*), there are two distinct values of *t*. Therefore, the curve relating *t* to *s* loops back to form a "double" curve with a single maximum (Fig 7). In order to simplify the fitting, we maintain the assumption from Section 4 that the water level never fluctuates beyond the points at which max(*s*_*P*_) and max(*s*) occur. With this restriction, one value of *s* now has exactly one value of *t* associated to it, so that we can fit the data with a single curve. This single curve is not related to the double curve described above but instead replaces it completely.

We believe that the curve of best fit is strictly increasing and concave-up. Indeed, if this is the case, then we have the following: Since *t* is concave-up, we have *t*″(*s*) > 0. Since *n* is linearly related to *t*, we have *n*″(*s*) > 0. Since *n*(*s*) = *s* + *f*(*s*), we have *f*″(*s*) > 0. Thus, the failure rate *f*_%_ increases as *s* increases. If *f*_%_ increases, then it is probable that *r* increases as *s* increases. Furthermore, *w* increases as *r* increases. Thus, the function that relates *s* and *w* is increasing, which is in fact the premise of our model. We can therefore regard the time-success data as the basis for our model. The converse, however, is not true; our model does not require that *t* be concave-up, although other curves will not increase at the rate necessary to fit the data. Thus, we use the concave-up exponential function to fit the data (*R*^2^ = 0.516, n = 69, p < 0.001), and we get the following (Eq 14):

$$t(s)=35.2e^{14.2s},\;1\leq s\leq \text{max}(s)$$ (14)

### 6. Deriving Number of Failures as a Function of Successes from Time-Successes Data

Given the relationship in Eq 14 between the number *s* of successful interactions made and the total interaction time *t*(*s*) required, it is possible to derive a relationship between *s* and the accompanying number of failures *f*(*s*) as follows: The time needed for a WF wasp to make one success is *t*(1). The value *s* = 1 indicates that the water level is *w* = 0 so that the number of failures accompanying *s* = 1 is *f*(1) = 0. Thus, the time required for a WF wasp to find and attempt its first interaction (success or failure) is *t*(1). Now, consider a WF wasp that makes *s* successes. This WF wasp makes *f*(*s*) failures and therefore attempts a total of *n*(*s*) = *s* + *f*(*s*) interactions. The total time required for a WF wasp to attempt *n*(*s*) interactions is *t*(*s*). We know from above that the first interaction takes time *t*(1). Thus, the time required for a WF wasp to leave the first CS wasp and to complete its interactions with the remaining *n*(*s*) − 1 CS wasps is *t*(*s*) − *t*(1). In this time, the WF wasp moves from one CS wasp to another *n*(*s*) − 1 times (the number of intervals between the *n*(*s*) CS wasps). Let Δ*t* (parameter 3) be the average time needed for a WF wasp to move from one CS wasp to another CS wasp and to attempt interaction with the latter. Since each of the *n*(*s*) − 1 intervals between the *n*(*s*) CS wasps takes time Δ*t*, we have (*n*(*s*) − 1)Δ*t* = *t*(*s*) − *t*(1). Solving for Δ*t* and noting that *n*(1) = 1 + *f*(1) = 1, we have the following (Eq 15):

$$\Delta t=\frac{t(s)-t(1)}{n(s)-n(1)}$$ (15)

The value of Δ*t* is an unknown constant that is independent of *s* and *w*. In Karsai and Wenzel [26], the average time between two successes was measured to be 9.6 seconds so that Δ*t* < 9.6. It is apparent from this equation that Δ*t* is the slope of the plot of the linear equation relating *t* and *n*.

Solving the above equation for *n*(*s*) yields:

$$n(s)=\frac{t(s)-t(1)}{\Delta t}+1=\frac{t(s)-t(1)+\Delta t}{\Delta t}$$

Since *f*(*s*) = *n*(*s*) − *s*, we have the following relationship between the number of successes *s* and the number of failures *f* that a wasp makes (Eq 16):

$$f(s)=\frac{t(s)-t(1)}{\Delta t}+1-s=\frac{t(s)-t(1)+\Delta -s\Delta t}{\Delta t}$$ (16)

Let us note for a later section that $f^{\prime \prime }(s)=\frac{t^{\prime \prime }(s)}{\Delta t}>0$ for all *s*. As in the case of *t*, the domain of *f* is 1 ≤ *s* ≤ max(*s*). In all of the above, the variable *s* can be replaced with *s*_*P*_ in the case of a PF wasp. This means that the relationship between successes and failures is the same for both a WF wasp and PF wasp.

We cannot use the function *f* to achieve numerical outputs unless we know the value of Δ*t*. If a point (*x*, *y*) such that *f*(*x*) = *y* is provided, then we can calculate Δ*t* and use this value to make *f* usable for numerical evaluations.

### 7. Equating Theoretical-Based and Empirical-Based Functions within Reasonable Error

Our theoretical model (Eqs 9–11) depends on two free parameters *σ* and *K*. The function (Eq 16) derived from empirical data depends on one free parameter Δ*t*. To find values for these parameters, we require that the theoretical and empirical functions agree with each other within reasonable error. We define the function (Eq 17):

$$E(S(w),r(w),K,\Delta t)=\frac{f(\bar{s}(w))-\bar{f}(w)}{f(\bar{s}(w))}=1-\frac{\bar{f}(w)}{f(\bar{s}(w))}$$ (17)

where $\bar{s}$ and $\bar{f}$ are Eqs 11 and 12, and *f* is Eq 16. Note that *S* and *r* both depend on *σ*. The function *E* gives the percent error between the theoretical number of failures $\bar{f}$ and empirically derived number of failures *f* for a given number of successes $\bar{s}$ at a specified water level *w* and under specified values of the model parameters. For theory and empirical functions to agree, we require |*E*| ≤ 0.05. That is, the percent error between *f* and $\bar{f}$ must be within 5%. This restriction will allow us to find the region of biologically feasible values for our model parameters.

To find the values of the model parameters, it is necessary to understand whether *E* increases or decreases with each of *r*, *K*, and Δ*t*. We observe by testing Eqs 10 and 11 that the following identity holds independently of *σ*, *K*, and Δ*t* (Eq 18):

$$r(w)=\frac{\bar{f}(w)}{\bar{s}(w)+\bar{f}(w)}$$ (18)

Thus, the resistance or probability of failure at a water level is equivalent to the colony-level failure rate as computed in terms of the average number of successes and average number of failures. In particular, if *r*(*w*) is increased, then we observe by testing Eqs 11 and 12 that $\bar{s}(w)$ decreases and $\bar{f}(w)$ increases. Since *f*′(*s*) > 0, decreasing $\bar{s}(w)$ implies decreasing $f(\bar{s}(w))$. Thus, by Eq 17, $\frac{\partial E}{\partial r}<0$. If *K* is increased, then we observe by testing Eqs 11 and 12 that $\bar{s}(w)$ and $\bar{f}(w)$ increase proportionately (since *r* is held constant). Suppose $\bar{s}(w)$ and $\bar{f}(w)$ increase by a factor *a* (*a* > 1) so that we have $E=1-\frac{a\bar{f}(w)}{f(a\bar{s}(w))}$. Since *f*″(*s*) > 0 (noted after Eq 16), *f*(*as*) = *bf*(*s*), *b* > *a*. Thus, $E=1-\left(\frac{a}{b}\right)\frac{\bar{f}(w)}{f(\bar{s}(w))}$, $\frac{a}{b}<1$. Hence, by Eq 17, $\frac{\partial E}{\partial K}>0$. If Δ*t* is increased, then $\bar{s}(w)$ and $\bar{f}(w)$ are unaffected (neither depends on Δ*t*) and $f(\bar{s}(w))$ decreases (by Eq 16). Therefore, by Eq 17, $\frac{\partial E}{\partial \Delta t}<0$. These findings will be used in the proceeding sections.

## Results

### 1. Bounds on and Approximate Values of the Model Parameters

In this section, we use our mathematical model to derive bounds on and approximate values of our model parameters. These values will then be used in proceeding sections to understand the relationship between the common stomach water level and the interactions that foragers attempt with CS wasps.

We first derive an upper bound on Δ*t*. Consider the water level *w* = 0.5. By definition, *r*(0.5) = 0.5 so that $\bar{s}(0.5)=\bar{f}(0.5)$ (by Eq 18). Thus, $n(\bar{s}(0.5))=\bar{s}(0.5)+\bar{f}(0.5)=2\bar{s}(0.5)$. By Eq 15, we have:

$$\Delta t=\frac{t(\bar{s})-t(1)}{n(\bar{s})-1}=\frac{35.2e^{0.142\bar{s}}-35.2e^{0.142}}{2\bar{s}-1}$$

Recall the restriction $1<\bar{s}(0.5)<5.8$ (Section 4). Because Δ*t* strictly increases with $\bar{s}\in [1,5.8]$, $1<\bar{s}<5.8$ implies 0 < Δ*t* < 3.74.

We now derive bounds on *σ*. Consider the water level *w* = 0.25. Recall that *S*(0.25) = 2 for all *σ* (Fig 3). Assume *K* > 0.1 (improving on *K* > 0) so that the probability that a WF wasp continues after a failure is at least 10%. Let *K* = 0.1 (its lower bound) and Δ*t* = 3.74 (its upper bound). Consider the equation *E*(2, *r*(0.25), 0.1, 3.74) = 0.05. Solving for *r*(0.25), we get *r*(0.25) = 0.170…. Since $\frac{\partial E}{\partial r}<0$, *r*(0.25) > 0.170… implies *E* < 0.05. To satisfy *E* > −0.05 (since we demand |*E*| < 0.05), we can have *K* > 0.1 or Δ*t* < 3.74 (since $\frac{\partial E}{\partial K}>0$ and $\frac{\partial E}{\partial \Delta t}<0$. On the other hand, *r*(0.25) < 0.170… implies *E* > 0.05. To satisfy *E* < 0.05 (since we demand |*E*| < 0.05), we must then have *K* < 0.1 or Δ*t* > 3.74, neither of which is possible. Thus, *r*(0.25) > 0.170…. This implies *σ* < 5.52…. Since we assumed *σ* ≥ 0 (Section 1.1), we have approximately 0 ≤ *σ* < 5.53. Note that this range of values is approximately the range for which *S*(0.5) = 6. That is, under a resistance function with a *σ* value in the deduced range, WF wasps must make six successful interactions to completely unload their water when the common stomach water level is *w* = 0.5.

We now derive bounds on *K* and a lower bound on Δ*t*. Assume to the contrary that *K* ≤ 0.957… (*K* = 0.957… is the value that results in *s*(0.5) = 5.2 under the deduced *S*(0.5) = 6). The restriction *K* ≤ 0.957 implies max(*s*) < 8 for all 0 < *σ* < 5.53; in the cases of both *σ* = 0 and *σ* = 5.53, we observe that *K* = 0.957 is not large enough to have max(*s*) > 8. We know, however, that the average number of successes that a WF wasp makes is 5.2 ± 2.8, which implies that max(*s*) > 5.2 + 2.8 = 8, which is a contradiction. Thus, *K* > 0.957…. Since $\bar{s}(0.5)$ increases with *K*, we have $\bar{s}(0.5)>5.2$. Also, *S*(0.5) = 6 and $\bar{s}(0.5)<5.8$ together imply *K* < 0.990…. Therefore, 0.957… < *K* < 0.990. Just as how $\bar{s}(0.5)<5.8$ implies Δ*t* < 3.74, $5.2<\bar{s}(0.5)$ implies 3.52 < Δ*t*. Hence, 3.52 < Δ*t* < 3.74; the average time between two attempted interactions (success or failure) is between 3.52 and 3.74 seconds.

We can use the bounds 3.52 < Δ*t* < 3.74 and 0.957… < *K* < 0.990… to strengthen the bounds on *σ*. Consider the inequality *E*(2, *r*(0.25), 0.957…, 3.74) < 0.05. By the same reasoning used in the second paragraph of this section, we get *r*(0.25) > 0.232…, which implies *σ* < 0.644…. We can improve this bound further. Recall *w*(*x*) is the water level at which Δ*w* = *x* (Eq 5), where Δ*w* = 1 / *S*. For every *S*, let *K*_min_(*S*) and *K*_max_(*S*) be the values of *K* solved from *E*(*S*, *r*(*w*(1 / *S*)), *K*, 3.52) = −0.05 and *E*(*S*, *r*(*w*(1 / *S*)), *K*, 3.74) = 0.05 respectively. For every *S*, we have the bounds *K*_min_(*S*) < *K* < *K*_max_(*S*). To satisfy these bounds for all *S*, we need max(*K*_min_(*S*)) < *K* < min(*K*_max_(*S*)). We observe that max(*K*_min_(*S*)) = *K*_min_(7) and min(*K*_max_(*S*)) = *K*_max_(2). Requiring the inequality *E*(2, *r*(0.25), *K*_min_(7), 3.74) < 0.05, we get *σ* < 0.516. This also implies *K* > *K*_min_(7) > 0.985… > 0.957…, where the exact value of *K*_min_(7) depends on *σ*. Since we still have *K* < 0.990…, we require 0.990…< *K*_max_(2) to guarantee that *K* can be arbitrarily close to 0.990…. The definition of *K*_max_(2) then implies *E*(2, *r*(0.25), 0.990…, 3.74) < 0.05, which yields *σ* < 0.494…≈ 0.494. Thus, 0 ≤ *σ* < 0.494.

Note that 0 ≈ 0.494 (e. g. *r*(0.25) ≈ 0.236 for *σ* = 0.494…). In particular, the average percent error between *r*(*w*) (*σ* = 0.494…) and *w* (*σ* = 0 is:

$$\int_{0}^{1}\left|\frac{r(w)-w}{w}\right|\text{d}w\approx 3.35\%$$

Thus, we approximate *σ* as *σ* = 0; that is, we conclude that the resistance function is approximately linear. The biological implications of this are mentioned in Results section 2. Now *σ* = 0 implies *K*_min_(7) = 0.986… so that approximately 0.986 < *K* < 0.990. Thus, the probability that a forager aborts its foraging cycle after any given failure is very low; out of every 100 failures observed (over any set of foragers), about 98 or 99 of them will be followed by another attempt at interacting, and 1 or 2 of them will be followed by an abortion of the foraging cycle. For every value of *K* between 0.986 and 0.990, Δ*t* seems to vary with the water level. However, Δ*t* varies strictly within its bounds 3.52 and 3.74. That is, although Δ*t* varies, it is always within 3% of 3.63, so that we can still regard Δ*t* as approximately constant.

### 2. Natural Bounds on the Number of Successes Needed for Complete Unloading of Water, and Interpreting the Linear Resistance Function

**Fig 8:**
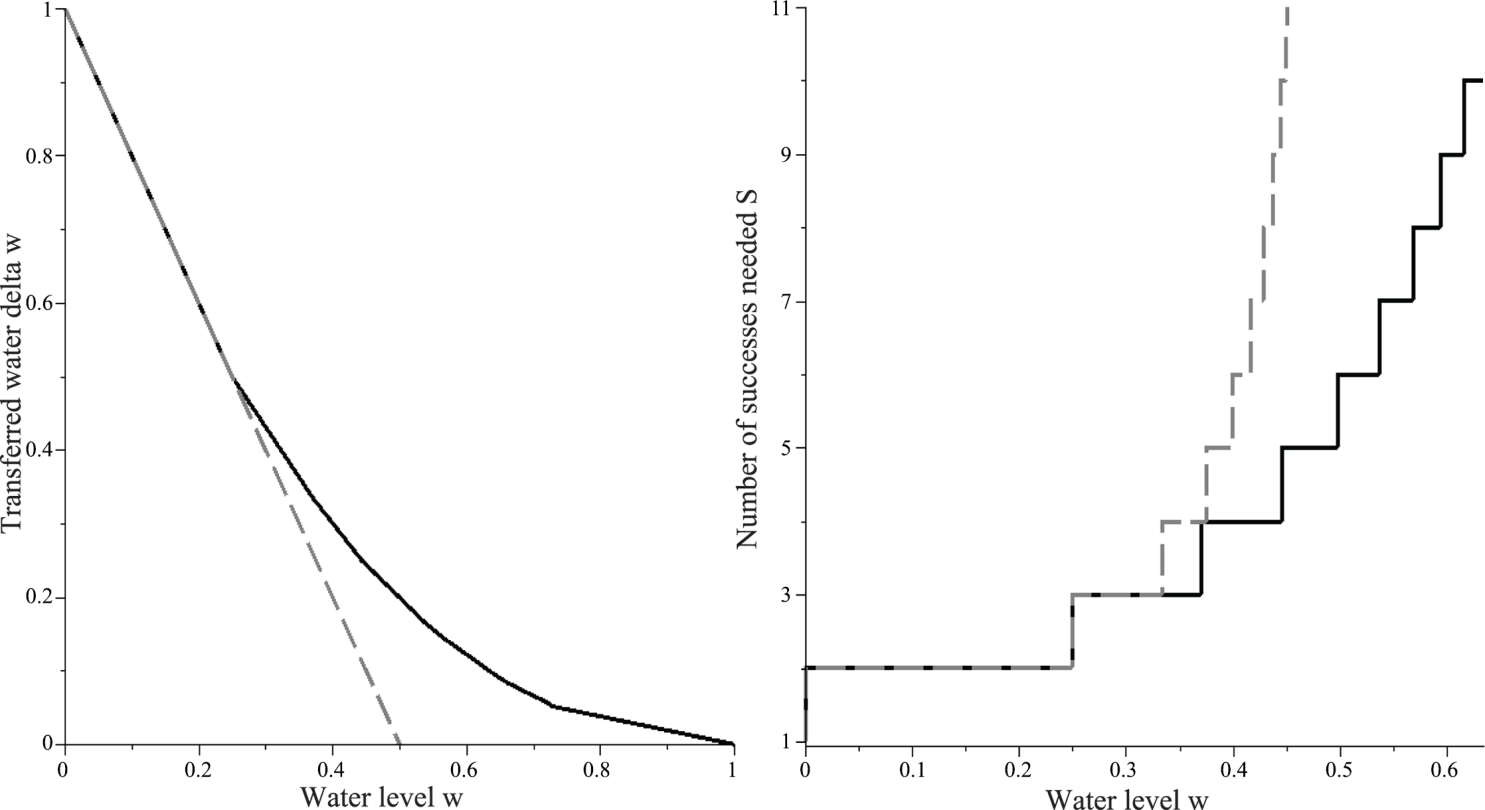
Lower and Upper bounds on the amount of water Δ*w* that a WF wasp is expected to transfer to an accepting CS wasp (8A) and on the number of successes *S* needed for a WF wasp to completely rid its load (8B) when the common stomach water level is *w*. These bounds follow from the bounds on the assumed resistance function: Resistance with *σ* = 0 (black solid line) defines an upper bound on Δ*w* and hence a lower bound on *S*. Resistance with *σ* = *∞* (grey dashed line) defines a lower bound on Δ*w* and hence an upper bound (for *w* < 0.5) on *S*.

As described in Section 1.2, the amount of water Δ*w* transferred from a WF wasp to a CS wasp and the number of successful transfers *S* needed for complete emptying are bounded both above and below. The bounding property results from the way in which Δ*w* depends on the resistance function (Section 1.2). The resistance function with *σ* = 0 results in an upper bound on Δ*w* and therefore a lower bound on *S*, while the resistance function with *σ* = *∞* results in a lower bound on Δ*w* and hence an upper bound on *S* for *w* < 0.5 (Fig 8). Thus, for nonzero water levels below 50%, a WF wasp must make multiple successful interactions (finite number) to completely rid its load. Although a half-full CS wasp has room to accept half of a crop load, it accepts less than this amount (Δ*w*(0.5) < 0.5). In particular, for *σ* = 0, a WF wasp must make 6 interactions to completely rid its load when the common stomach is half-full. Thus, a linear resistance function might explain why foragers make on average about 5–6 successful interactions. Although Δ*w* under *σ* = *∞* is undefined for *w* > 0.5, *r*_∞_(*w*) = 1 for *w* > 0.5; since a WF wasp is guaranteed to be rejected by a CS wasp with *w* > 0.5 (under *σ* = *∞*), no water is transferred. A resistance function with a high value of *σ* is sensitive to changes in water level about *w* = 0.5 and encourages water in-flux at small *w*. Thus, even under *r*_∞_, a non-zero value of Δ*w* is expected for *w* < 0.5. This result is significant because there are natural restrictions on the number of successful interactions needed regardless of *σ*; these bounds are independent of empirical data and is built into the system. Consider, for example, the water level *w* such that *s*(*w*) = 5.2. This implies *S*(*w*) > 5.2. Since *S*_∞_(*x*) > *S*(*x*) for 0 < *x* < 0.5, *S*_∞_(*w*) > 5.2. By Eq 8, this is $\text{ceil}\left(\frac{1}{1-2w}\right)>5.2$, or $\frac{1}{1-2w}>5$. Solving for *w*, we get *w* > 0.4. Thus, a WF wasp can make 5.2 successes only at water levels greater than 40%. As another example, in our model, an intrinsic feature of the system is that at *w* = 0.5, at least six successes are needed for a WF wasp to completely empty itself, guaranteeing multiple interactions.

In this context, the result that *σ* ≈ 0 (Results Section 1) is significant because it implies that *S*(*w*) is minimized for every *w*; *σ* = 0 minimizes the number of successes needed for complete unloading, and therefore maximizes the efficiency of water transfer. Note that while water transfer efficiency is maximized, the lower bound on *S* is such that multiple interactions are still necessary for complete unloading. Thus, as our model suggests, the system may have evolved such that the necessity for multiple interactions is built-in but also minimized within that constraint.

**Fig 9:**
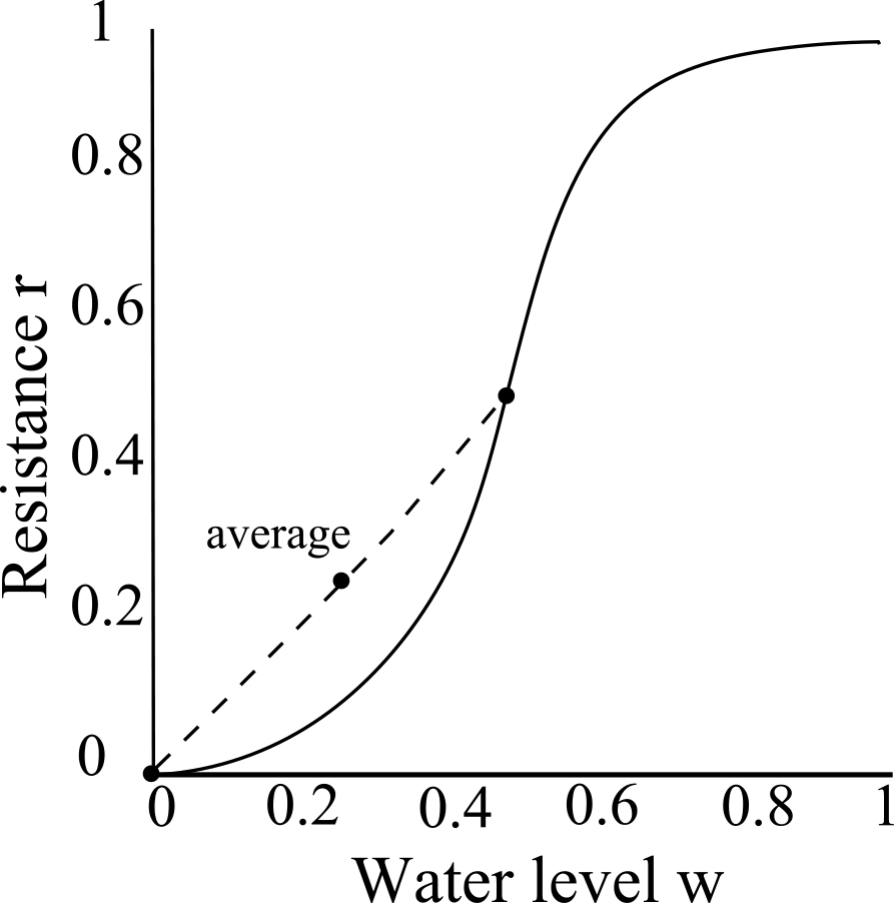
Schematic plot of how nonlinear individual resistance functions *r* averaged over different water levels of the common stomach *w* might yield a linear average resistance function. Suppose individual CS wasps have sigmoid resistance functions (black solid line). Suppose half of the CS wasps are empty (*r*(0) = 0) and the remaining half are half-full ((*r*(0.5) = 0.5). The average resistance of the whole common stomach lies on the secant line (grey dashed line) joining (0, 0) and (0.5, 0.5); the average water level is *w* = 0.25, and the average resistance is *r* = 0.25. This means that a linear average resistance function does not imply linear individual resistance functions. Thus, the result that *σ* = 0 holds for the common stomach as a whole but does not necessarily hold for individual CS wasps.

Because we assumed that all CS wasps have the same water level (Section 1.1), we interpret the resistance function as the average resistance supplied by the common stomach as a whole. Thus, since *σ* = 0, the average resistance function is approximately linear. This does not imply that the resistance function of every individual is necessarily linear; it is possible that resistance values from different water levels with *σ* > 0 average to a linear function (Fig 9). Suppose there is variance in the distribution of water among CS wasps. Suppose, for example, that 50% of the CS wasps have *w* = 0, and the other 50% of the CS wasps have *w* = 0.5. The average water level of the common stomach is then 0.25, and the average resistance is $\frac{r(0)+r(0.5)}{2}=\frac{0+0.5}{2}=0.25$. Thus, on average, *r*(0.25) = 0.25, regardless of *σ*. Therefore, the system may be minimizing *S*(*w*) for every *w* by setting the water distribution among CS wasps such that the average resistance function is linear. This allows for the possibility that (1) the individual resistance function has *σ* > 0 and that (2) individual resistance functions may vary among CS wasps (*σ* may vary from CS wasp to CS wasp). By (1), the system can be both a strong buffer (by individuals) and could still allow for the easy flux of water (on average). By (2), an exact value of *σ* for individual resistance functions might not be critical so long as these individual functions average to a linear function.

### 3. Performance of Foragers Predicted by the Model

**Fig 10:**
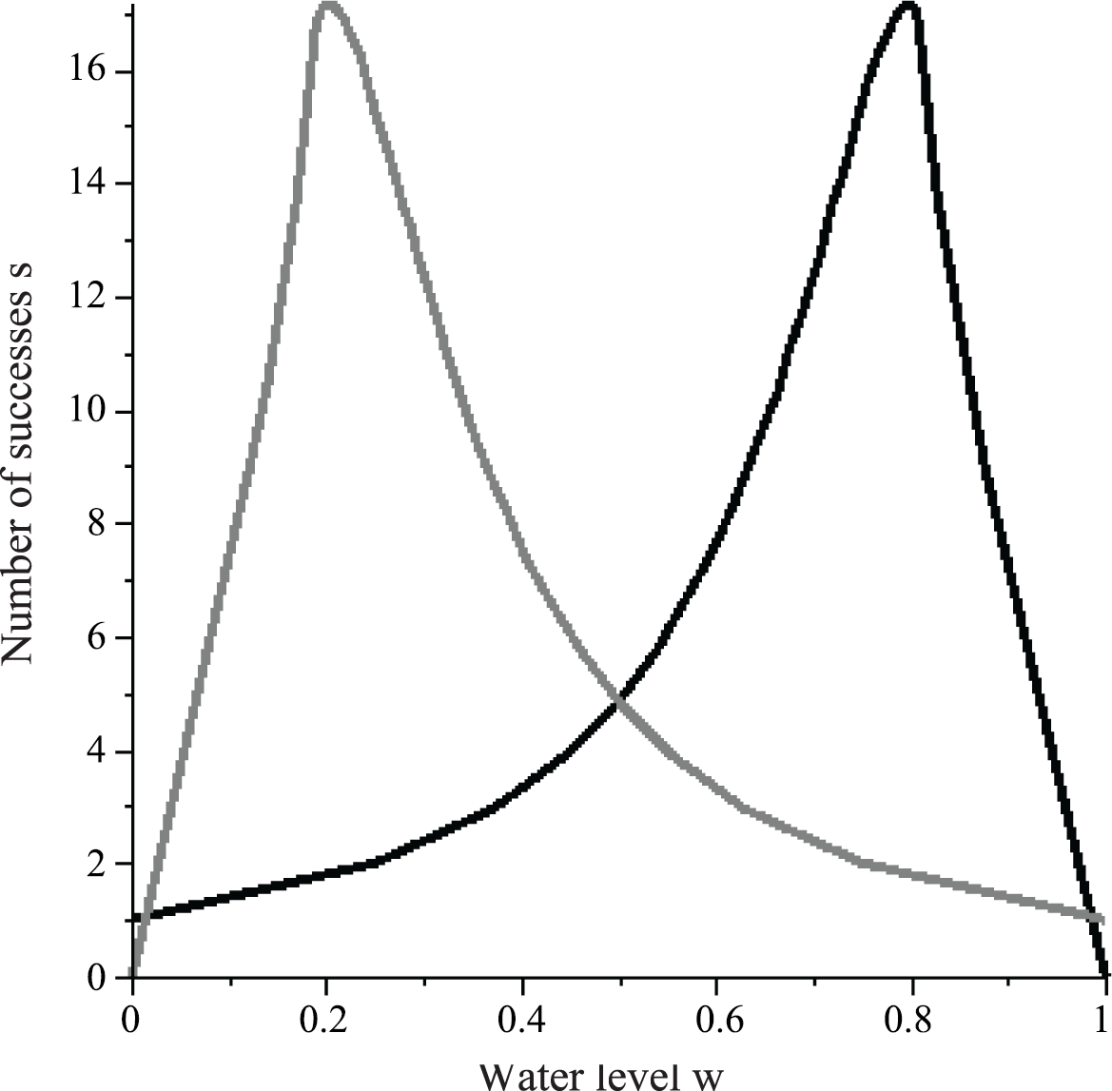
Plot of the numbers of successes *s* and *s*_*p*_ that a WF wasp (black) and PF wasp (grey) are expected to complete in one foraging cycle when the common stomach water level is *w*. The plots are based on *σ* = 0 and *K* = 0.988 (Results Section 1). The plot for the WF wasp is an approximation obtained by joining the right endpoints of a step function. The plot for the PF wasp is symmetric to the plot for the WF wasp about the line *w* = 0.5.

In this section, we first examine the approximate number of successes *s* and *s*_*P*_ that a WF wasp and PF wasp are expected to make respectively when the common stomach water level is *w* (Fig 10).

Since *S* (number of successes needed) is an integer while resistance *r* and the parameter *K* are real numbers, the plot of *s*(*w*) should be step-like. It is, however, sufficient for our analysis to consider only the plot of the right end-points of the steps of *s*(*w*). Note that *s* = 5.2 occurs at water level *w* > 0.5 (Fig 10), which is a consequence of our simplification of the plot of *s*(*w*). Since *s*_*p*_(*w*) = *s*(1 − *w*), the plot for *s*_*p*_(*w*) is obtained as the reflection of the plot for *s*(*w*) over *w* = 0.5. In general, the shapes of *s*(*w*) and *s*_*p*_(*w*) agree with their hypothesized shapes (Fig 5).

Now let *w*(*x*) be the water level such that *s*(*w*(*x*)) = *s*; that is, let *w*(*x*) be the water level at which a WF wasp is expected to make *x* successes. We see that *w*(5.2) = 0.514 and *w*(6.4) = 0.568. So, 1 − *w*(6.4) = 0.438, which is the water level at which *s*_*p*_ = 6.4 (Fig 10). We can substitute these values into Eq 13 to achieve the following bounds on the average water level $\bar{w}$:

$$0.438<\bar{w}<0.476$$

Hence, the average water level has a value slightly less than 50%. Although this was qualitatively derived in part from the empirical observation that WF wasps and PF wasps make on average 5.2 and 6.4 successes respectively [26], we used our model to determine at what water levels these average values occur. We can now conclude the following: The average water level is slightly less than 50%, and because the resistance function is linear and WF wasps are very unlikely to abort their foraging cycles (large *K*), a particular relationship between *w* and *s* emerges. This relationship is also founded on the precise way that resistance and amount of water accepted by a CS wasp are related (Section 1.2). Under this precise relationship, the water level fluctuates about its average value such that we observe the emergent averages 5.2 and 6.4. Thus, the values 5.2 and 6.4 as average numbers of successes are indicators of more fundamental parameters that underlie the system.

We now observe that *y* = *s*(*w*) has a maximum value of 17.158 at *w* = 0.800 (Fig 10). This implies that *y* = *s*_*p*_(*w*) has the same maximum value at *w* = 0.200. It is not necessary that the common stomach water level ever reaches the value *w* = 0.200 or *w* = 0.800. So, at most, 17.158 is simply an upper bound on the maximum number of successes that a forager is expected to make. For the same reason that the average number of successes is greater for PF wasps than for WF wasps (since $\bar{w}<0.5$), the maximum number of successes should be greater for PF wasps than for WF wasps. In data collected from real colonies, the largest value of *s* is 12, and the top two largest values of *s*_*p*_ are 15 and 19 (Fig 6) [26]. Thus, our result that *s*_*p*_ ≤ 17 is approximately accurate for PF wasps. Note that in the case of *K* = 0.990 (the upper bound on *K*), the maximum number of successes predicted is 19. So, let max(*s*_*p*_) = 17, and for WF wasps, let max(*s*) = 12. We see that *w*(12) = 0.694 (Fig 10), and we already showed that 1 − *w*(17) = 0.200. If the common stomach water level fluctuates symmetrically about the average water level $\bar{w}$, then $\bar{w}$ is the midpoint of 1 − *w*(17) and *w*(12): $\bar{w}=0.447$. Note that this is consistent with the bounds we obtained on $\bar{w}$ in the previous paragraph.

**Fig 11:**
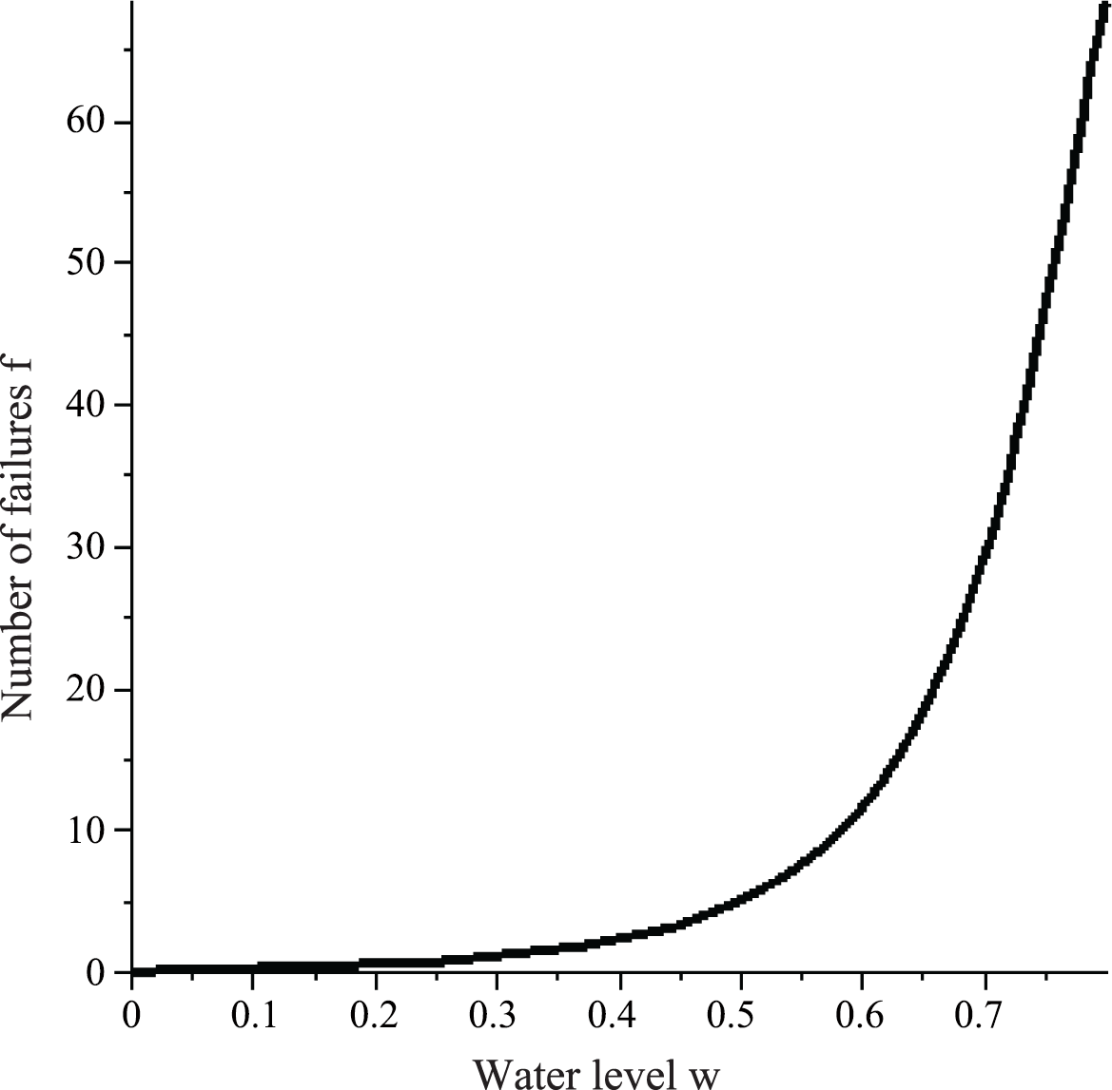
The number of failures *f* that a WF wasp is expected to make in one foraging cycle when the common stomach water level is *w*.

The number of failures *f* that a WF wasp is expected to make increases with the common stomach water level *w* (Fig 11).

Note that the plot of failures for a PF wasp can be obtained by reflecting *y* = *f*(*w*) about *w* = 0.5. Because we showed that the common stomach water level fluctuates between the water levels at which a PF wasp and WF wasp are expected to make their maximum numbers of successes (*w* = 0.200 and *w* = 0.694 respectively), it is sufficient to plot *f*(*w*) only for *w* ≤ 0.800; we include water levels between 0.694 and 0.800 because the reflection of this region is included in the plot for a PF wasp. We see that as the common stomach water level increases, a WF wasp is expected to make more and more failures. The maximum number of failures that can be expected is about 71 at about *w* = 0.8 compared to 30 at *w* = 0.694. Although 71 is large, such extreme water levels are probably infrequent. Such large values, however, help to explain the relationship between number of successes and handling time; handling time appears to increase exponentially with success number, and this is because increasing success number implies increasing water level (Fig 10) which in turn implies increasing failure number (Fig 11). Since $r(w)=\frac{f(w)}{s(w)+f(w)}$, $f(w)=\frac{r(w)s(w)}{1-r(w)}$, and since *r*(*w*) = *w* (*σ* = 0), $f(w)=\frac{s(w)w}{1-w}$. Thus, *f*(*w*) increases with *w* at a faster rate than does *s*(*w*). Given that *f* is concave-up, we expect the average value of *f* to be greater than the value of *f* at $\bar{w}=0.447$ and at *w* = 0.476. Thus, the average number of failures is greater than *f*(*w* = 0.476) = 4.1. Further, depending on the concavity of *f*, it is possible that its average value could occur at a water level *w* > 0.514, where *w* = 0.514 is the water level at which the average number of successes (5.2) is made. Hence, more failures on average than successes does not necessarily imply that $\bar{w}>0.5$, since it is also consistent with $\bar{w}<0.5$. The possibility that more failures are made on average than successes is left open.

If *σ* is increased from 0, then *S*(*w*) increases for all *w* > 0 (Fig 3 and Results Section 2) and *r*(*w*) decreases for 0 < *w* < 0.5. From $f(w)=\frac{r(w)(s)}{1-r(w)}$, it is unclear whether *f*(*w*) increases or decreases with *σ* for *w* < 0.5. We expect, however, that *f*(*w*) decreases for *w* < 0.5 since *r*(*w*) → 0 for *w* < 0.5 as *σ* → *∞*. This decrease in failures, however, will not significantly increase the efficiency of water transfer since we will see that WF wasps are able to unload most of their water at low water levels even under *σ* = 0. On the other hand, for *w* > 0.5, as *σ* is increased, we expect *f*(*w*) to increase since both the number of successes needed *S* and the resistance *r* increase for *w* > 0.5. This means that *σ* = 0 minimizes the number of failures expected for a WF wasp at water levels greater than 50%. Although this seems to contradict the idea that negative feedback in the form of failures should be larger for *w* > 0.5, we see that the expected number of failures is large even for *σ* = 0. Thus, it seems that larger failure expectancy would not benefit the system significantly and that the minimum number of failures, which is itself large for high water levels, is sufficient. Further, this reinforces the fact that the common stomach is not only a buffer but also a mediator of water between WF wasps and PF wasps; if water flux was over-restricted by a very large *σ* value, then the system would not be an efficient mediator of water. The value *σ* = 0 might be able to provide both strong negative feedback and still allow for efficient mediation of water. Also, as mentioned in Results Section 2, it is possible that the individual resistance function is stronger than the average resistance function, so that the plot of failures might be steeper for individual CS wasps.

**Fig 12:**
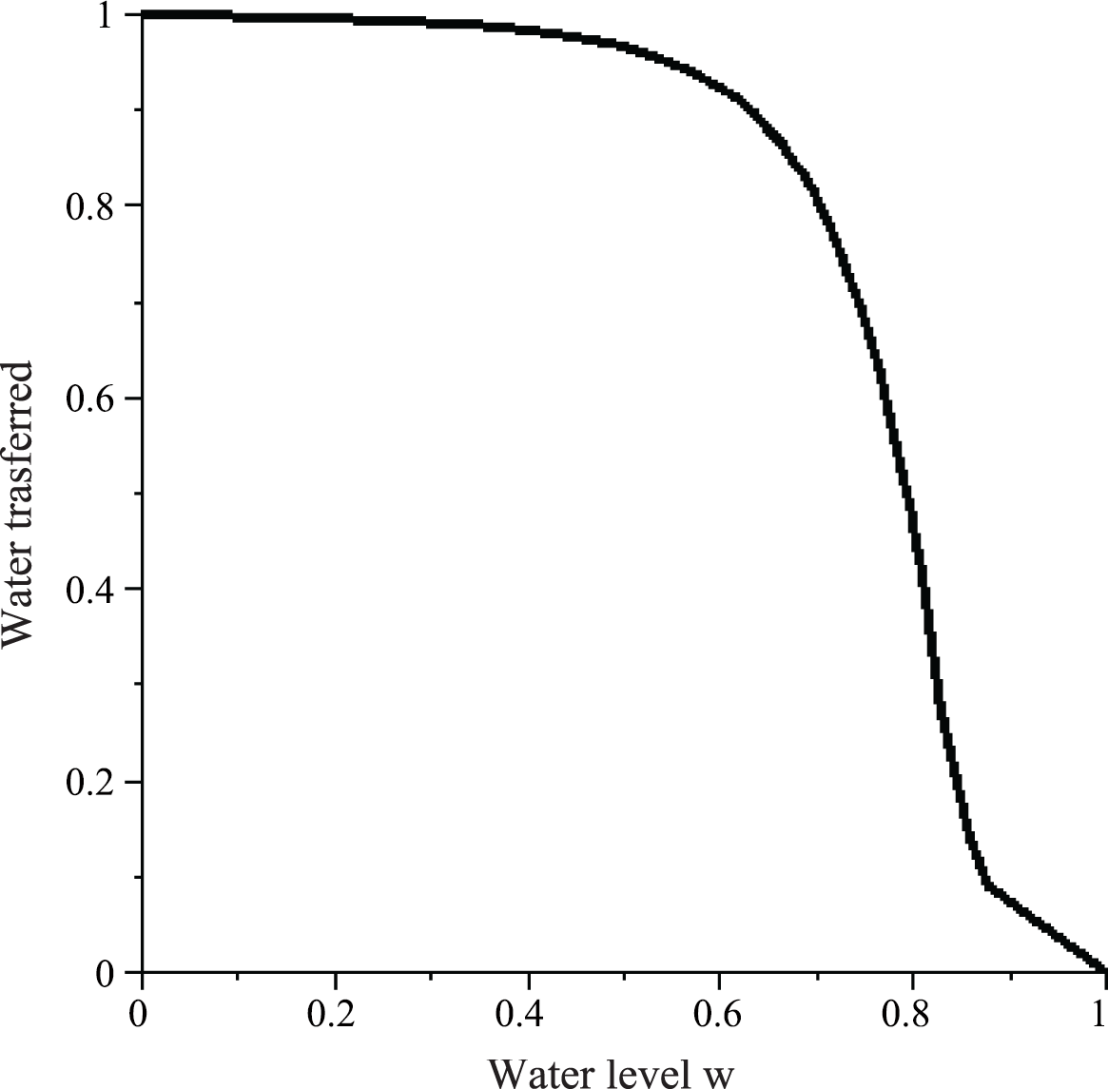
The fraction of one crop load of water that a WF wasp is expected to successfully transfer in one foraging cycle when the common stomach water level is *w*.

We now examine the fraction of one crop load of water that a WF wasp is expected to unload in one foraging cycle when the common stomach water level is *w* (Fig 12). Recall that one foraging cycle begins when the WF wasp arrives with a full load of foraged water and ends when the WF wasp either empties its crop or aborts the cycle. In one foraging cycle, when the water level is *w*, a WF wasp transfers Δ*w*(*w*) of its crop per successful interaction and makes *s*(*w*) such successes. Thus, the total amount of water (fraction of one crop) that the WF wasp successfully unloads is *s*(*w*)Δ(*w*). By Eq 7, this is approximately $\frac{s(w)}{S(w)}$ (completed fraction of successes needed for complete emptying). Note that for PF wasps, the amount of water that a PF wasp is able to receive in one foraging cycle at water level *w* is $\frac{s(1-w)}{S(1-w)}$. We see that for most water levels, a WF wasp is expected to unload almost all of its water into the common stomach (Fig 12). Around *w* = 0.7, the fraction of unloaded water begins to decrease rapidly. This is because although *S*(*w*) continues to grow at *w* = 0.7 (Fig 8), *s*(*w*) begins to plateau to its maximum at *w* = 0.7 (Fig 10). For *w* > 0.8, the WF wasp is not able to unload much of its water; it aborts its job due to the high resistance from the CS wasps.

We want to focus on the result that a WF wasp unloads approximately 50% of its load when *w* = 0.8, which is approximately where the plot of *s*(*w*) has its maximum value. Although we said that the water level remains strictly below *w* = 0.694, it is still relevant to study the plots at *w* = 0.800 because we are equating the behavior of a WF wasp at *w* = 0.800 to the behavior of a PF wasp at *w* = 0.200, and the water level can reach *w* = 0.200.

The result that WF wasps are expected to unload about half of their load at the water level where *s*(*w*) has its maximum value is significant because it might provide a biological explanation for the evolution of the value of the parameter *K*. Suppose we decrease *K* below 0.988. Since this increases the probability of abortion, it decreases the number of successes expected for a forager; *s*(*w*) decreases for all *w*. The function *S*(*w*), however, is independent of *K*. Thus, decreasing *K* decreases $\frac{s(w)}{S(w)}$. Since there exist water levels for which $\frac{s(w)}{S(w)}=0.5$ under *K* = 0.988, values of *K* below 0.988 will imply some range of water levels over which WF wasps unload less than half of their crop loads. It could be possible, however, that the system evolved such that WF wasps and PF wasps can be expected to unload and fill respectively at least half of their crops at every common stomach water level within its fluctuating range. Thus, it is possible that *K* = 0.988 evolved to ensure a certain minimum on the average amount of unloading (for WF wasps) and loading (for PF wasps). If *K* is increased above 0.988, then WF wasps will be expected to unload more than 50% at all water levels below *w* = 0.8. This does not, however, provide any advantage; at low water levels, WF wasps are already efficient (Fig 12), and at higher water levels, increasing the in-flux of water by increasing *K* is counter to the negative feedback of the resistance function. Thus, *K* = 0.988 ensures that foragers are "at least" 50% successful with their task at all feasible water levels. This idea is explained in greater detail in the next section.

**Fig 13:**
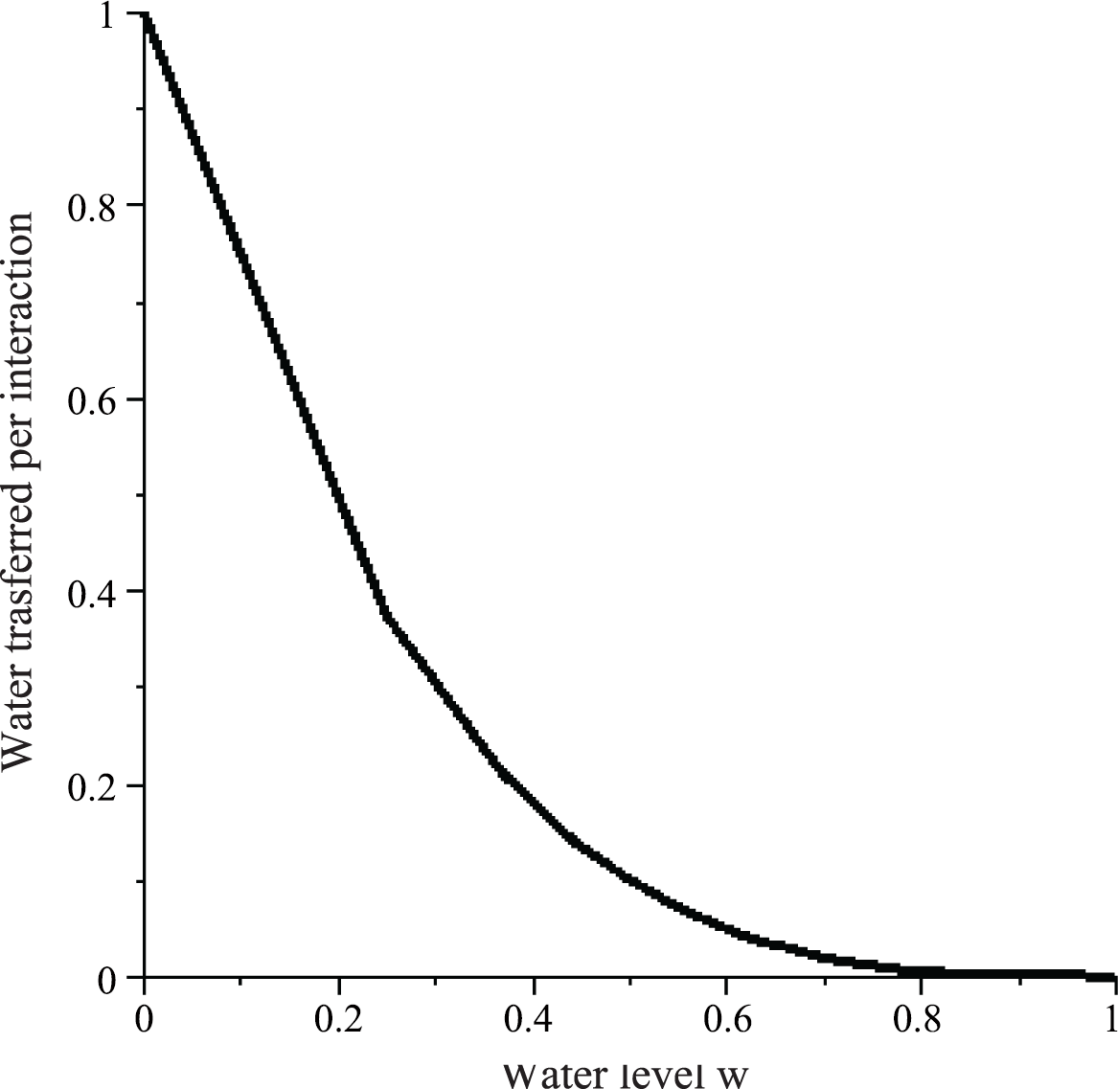
The amount of water that a WF wasp is expected to unload per interaction (normalized) when the common stomach water level is *w* (the amount of water is given as a fraction of one crop load). This is the rate at which water is transferred into the common stomach per interaction: $\frac{1-r(w)}{S(w)}$.

Finally, we examine the fraction of one crop of water that a WF wasp is expected to transfer on average per interaction (success or failure) when the common stomach water level is *w* (Fig 13). Since $\frac{s(w)}{S(w)}$ is the fraction of one crop that a WF wasp is expected to unload in one foraging cycle and since one foraging cycle contains *s* + *f* interactions, the average amount of water transferred per interaction is $\frac{s}{S(s+f)}$. This is $\frac{1}{S}\left(1-\frac{f}{s+f}\right)$ = $\frac{1}{S}\left(1-r(w)\right)$ = $\frac{1-r(w)}{S(w)}$. Thus, when the common stomach water level is *w*, a WF wasp is expected to unload water at a rate of $\frac{r(1-w)}{S(w)}$ crop loads per interaction (this value must be a fraction of one crop load). Since we are assuming that each interaction, including the time needed to move from one CS wasp to another, takes an average time of Δ*t*, the rate of transfer per interaction is a measure of the time rate of water unloading. For PF wasps, the rate at which water is expected to be transferred to a PF wasp per interaction is $\frac{r(w)}{S(1-w)}$. We see that $\frac{1-r(w)}{S(w)}$ decreases with *w* (Fig 13). Since *σ* = 0, we have $\frac{1-w}{S(w)}$, and we notice that 1 − *w* decreases with *w* while *S*(*w*) increases with *w*. Thus, the decreasing trend is mathematically expected. Biologically, the rate at which a WF wasp unloads water per interaction decreases as the common stomach water level increases. Note that this rate does not depend on the parameter *K* since neither *r* nor *S* depends on *K*. Since both *r* and *S* depend on *σ*, and since the value of *σ* is probably "encoded" into the behavior of CS wasps and not foragers (even if the *σ* value resulted from system-level interactions), the rate of loading and unloading per interaction is determined by the common stomach. For *w* = 0.25, if *σ* is increased, then *S* remains the same, *S*(0.25) = 2 (Fig 3), while 1 − *r*(0.25) increases. Thus, if *σ* is increased, then the rate at which water flows into the common stomach is increased at *w* = 0.25. We expect a similar trend for other water levels near *w* = 0.25. Thus, it seems that the system would be more efficient if *σ* was larger, but we found that *σ* is set at its minimum value of 0. Part of the resolution to this question might be as follows: At *w* = 0.5, if *σ* is increased, then *r*(*w*) remains the same since *r*(0.5) = 0.5 for all *σ*, while *S*(0.5) increases with *σ*. Thus, if *σ* is increased, then $\frac{1-r(0.5)}{S(0.5)}$ will decrease for both WF wasps and PF wasps by symmetry. Thus, since the common stomach water level is probably *w* = 0.5 more frequently than it is *w* = 0.25, the system may have evolved so that the flow rate of water in and out of the common stomach per interaction is maximized at or near *w* = 0.5. In particular, since *S*(0.5) = 6 for *σ* = 0, this maximum flow rate is $\frac{1-0.5}{6}=\frac{1}{12}$ crop loads per interaction. This maximum flow rate should maximize the efficiency with which the common stomach mediates water from WF wasp to PF wasp, while it seems that the resistance function is still "strong" enough to regulate the common stomach water level from fluctuating too much.

## Discussion

The aim of our study was to understand by mathematical reasoning the feedback mechanisms regulating task partitioning of social wasps [7, 26] and to point out important characteristics of the "common stomach", which is in the center of this regulation mechanism [26, 31,30, 35, 36, 37]. These feedback mechanisms should in part determine the number of successful interactions that foragers are expected to complete, which in turn influences task switching. But instead of focusing on task switching as we did in our previous models, here we explained the functioning of the common stomach itself. Moreover, by assuming that every CS wasp is identical, we could reduce our analyses to individual forager-to-CS wasp interactions (i.e., regulation of water transfer at the individual level). We addressed in detail 1) why WF wasps and PF wasps make on average 5.2 and 6.4 successes per foraging cycle, 2) why there is a large variance in the number of successes that foragers make, 3) why the common stomach stabilizes at a given water level, and 4) how we can calculate some important parameters that are fundamental to the regulation of the system (e.g., strength of resistance and probability of abortion).

Ratnieks and Anderson [33] and Hart and Ratnieks [34] showed that multiple transfers of materials between individuals in a task partitioned system improve information acquisition. In our field study [26], we found that foragers make on average 5–6 successful transfers in a single foraging cycle and therefore we provided support for the evolutionary hypothesis of Ratnieks and Anderson [33]. However, in the current paper, we explain why the numbers of successful interactions are in fact 5.2 for WF wasps and 6.4 for PF wasps on average. Our goal was to provide a mechanistic (not evolutionary) explanation of the nature of multiple interactions, and therefore we focused on individual interactions and not colony-level optimization.

The multiple interactions, as we show, are the consequence of a series of successful and unsuccessful material transfers. While the multiple interactions evolved into important information for the colony, the mechanism itself stems from simple rules and constraints at individual levels. Real colonies show a large range (from 1 to 12 for WF wasps) in the numbers of successful material transfers after a single collecting trip [26], which can be explained without assuming that this variation is a characteristic of the WF wasps. Let us assume, as we did in our model, that all WF wasps that arrive at the common stomach are identical in that they all carry one crop load of water. Under this assumption, we do not expect a significant variance in the behaviors of these WF wasps. If optimizing accuracy would require 5–6 successes, why do some WF wasps make only one success or some much more? Although there is no variance in WF wasps to explain the variance in the numbers of successes, there is variance in the water level of the CS wasps. Thus, the number of successful transfers that a forager will perform must depend on the common stomach water level, which implies that the fullness of individual CS wasps influences the number of successes and therefore also the number of interactions.

The common stomach not only provides an information center for the colony and an interface to download and upload water, but it is also important as a buffer for the system [26]. Therefore it is logical to assume the existence of a negative feedback loop regulating the flux of water into and from the common stomach. At the individual level, we described this by a simple function, which is used by a CS wasp based on its own state instead of the state of the common stomach. Based on its own water level, a given CS wasp can 1) refuse to accept water or 2) accept only a fraction of a crop load of water from the WF wasp. It is also reasonable to hypothesize that a CS wasp with more water will accept less water if it accepts at all. Hence, we defined the resistance function *r* and the fraction of one crop load that a CS wasp is willing to accept Δ*w*. Now, since Δ*w* is the fraction of one crop load that a WF wasp is able to transfer per success, the reciprocal of Δ*w* is the number of successes *S* needed for the WF wasp to rid its entire load. However, WF wasps will "fail" to transfer water to those CS wasps that refuse to accept water (because they are close to full). Therefore, as it is observed in real colonies [26], a fail-safe mechanism ensures that the WF wasp will abort the foraging cycle if it is unable to transfer all of its water after a large number of interactions. We defined *K* to be the probability that a forager does not abort after a failure. The parameter *K* along with the number of successes *S* (needed for complete transferring of water) and the resistance function *r* are sufficient for determining the average number of successful interactions $\bar{s}$ that a WF wasp is expected to actually complete at a given water level of the common stomach *w*.

Our previous models [28, 30, 29, 31] used simple linear or sigmoid shaped functions for triggering the decisions for the wasps to abort a task in which they were engaged. In the current model, instead of using predetermined functions, we actually derived these decision functions from the elementary interactions between the individuals. In particular, we hypothesized the general shapes of these functions but used empirical data and additional assumptions to calculate the parameters determining the precise shapes. A key feature of our model is that we unified *r*(*w*) and *S*(*w*) with a single parameter *σ* (Section 1.2), which gives the curvature or strength of the resistance function, where the resistance function governs water transfer with individual CS wasps. Our central hypothesis is that a CS wasp with water level *w* accepts Δ*w*(*w*) of one crop load such that Δ*w*(*w*) "generates" the water level 1 − *w*; by "generating," we mean that consecutively transferring Δ*w* into an empty crop of the wasp until the crop "refuses" to accept more (by the resistance function) should give a terminal water level of 1 − *w*. An equivalent but more biological way to picture this is the following: Suppose we have a full CS wasp, and suppose it transfers water to a string of PF wasps by an average amount Δ*w* per PF wasp. Eventually, the CS wasp might resist and not transfer water to a PF wasp, and suppose by this point the CS wasp has water level *w*. According to our hypothesis, this CS wasp with water level *w* will accept precisely the amount Δ*w* from a WF wasp that it was giving away per PF wasp. It is possible to use this principle to get an explicit form of Δ*w*(*w*) and *S*(*w*).

Note that this relationship depends on the crucial assumption (which we examined also in detail) that there exists a symmetry between WF wasps and PF wasps; the interaction between a WF wasp and a CS wasp is identical to the interaction between a PF wasp and CS wasp, except for the direction of water transfer. In fact, this symmetry is perhaps the essence of our model. An immediate consequence of this hypothesis is that there are natural bounds on the number of successes that a WF wasp needs to make, which follow from natural bounds on the resistance function (Section 2). The lower bound on *S* implies that WF wasps and PF wasps must make 6 successful interactions to completely empty and fill their crops respectively. This agrees with the average value of successful interactions observed in nature [26]. This is especially noteworthy given that our mathematical derivation did not use observational data where this was derived. Thus, it might be that the average number of successes made is a consequence of a much more fundamental fact about the structure of the common stomach.

Once we formulated the numbers of successful and failed interactions as functions of the common stomach water level, the next natural step was to deduce the values of the model parameters *σ* and *K* that fitted empirical observations [26]. These values would help us understand exactly what properties of the system determine the dependence of successes on water level and why a particular form of dependence evolved. We concluded that the empirical observation that PF wasps needed more successful interactions on average than did WF wasps (6.4 > 5.2) [26] for finishing a foraging cycle implied that the average common stomach water level was less than 50%. In fact, in all our previous models [28, 30, 31] we observed the emergence of a stabilized common stomach below 50% fullness. In this paper, we were able to explain why the water level in the common stomach is on average stabilized below 50%. By the assumed symmetry of how CS wasps interact with WF and PF wasps, the mechanisms regulating the flux of water through the common stomach should drive the water level to 50%. A "leak" in the common stomach due to water consumption could lower the water level below 50%. In real colonies, observations show that water is indeed sometimes used for reasons other than pulp foraging—e.g., for cooling [26]. Nevertheless, we can still hold on to the assumption that a CS wasp interacts with WF wasps and PF wasps in a symmetric way. This way we explained why the water level is below 50% and hence why PF wasps make more successes than do WF wasps without complicating the CS-forager interactions. This allowed us to derive additional empirical constraints on the function relating successes to water level, e. g. *s*(0.5) < 5.8 (Section 4).

Queuing delays are important signals to insect societies for bottleneck of the partitioned tasks [33, 38, 27]. At the individual level, these delays stem from the extra time engendered by failed interactions. Using field data of Karsai and Wenzel [26] (Fig 6), we showed that the number of failures that accompany a given number of successes must increase with successes at an increasing rate, which again suggested that successes increase with water level since resistance increases with water level. Further, we inferred the number of failures for each number of successes by assuming that the time needed to finish a task must vary linearly with the total number of interactions, since we assumed that the average time Δ*t* between any two interactions is a constant. Using this requirement along with our earlier mathematical derivations, we were finally able to determine the values of the model parameters Δ*t*, *σ*, and *K* (Section 7). We found that 1) the average time between any two interactions, including the time for one of these interactions itself, is Δ*t* = 3.63 ± 0.11 seconds, 2) the resistance function is approximately linear (*σ* = 0), and 3) the probability that a forager will attempt another interaction after a failure is *K* = 0.988 ± 0.002. To explain the value of *K*, we recall our derivation that at all feasible common stomach water levels, WF wasps and PF wasps unload and fill up respectively at least half of their crops over one cycle of interactions (Fig 12). We showed that *K* > 0.988 provides no additional advantage and that *K* < 0.988 is less effective at some water level of the common stomach (Results Section 3). If we assume that specialist foragers should be maximally effective, then those individuals should satisfy a certain minimum level of success, such as (for WF wasps) unloading at least 50% of a crop load and (for PF wasps) filling at least 50% of a crop in one interaction. Such a threshold requires the foragers to attempt another interaction after a failure *K* = 98.8% of the time.

To explain the linearity of the resistance function (*σ* = 0), we have to consider the rate at which water enters the common stomach; this rate is given by the difference in the rates at which a WF wasp deposits water and a PF wasp withdraws water:

$$\frac{r(1-w)}{S(w)}-\frac{r(w)}{S(1-w)}.$$

If *σ* is increased, then the absolute value of this rate increases; for water levels below 50%, WF wasps deposit water at a greater rate and PF wasps withdraw water at a lesser rate, and at water levels above 50%, the opposite is the case (Results Section 3). The effect of inequalities in water download and uptake was also studied in detail in Hamann et al. [35].

Thus, if *σ* is increased, the force with which the resistance function drives the water level back to 50% increases. The result that *σ* = 0 therefore minimizes the strength of the resistance function and therefore maximizes the range of fluctuation of the common stomach water level. Note that *σ* = 0 also minimizes failures for water levels above 50% (for WF wasps) and consequently maximizes the water level at which the maximum number of successes occurs. Since CS wasps accept/transfer the most water at extreme water levels, the ease with which the water level can fluctuate under a linear resistance function can allow for optimal mediation of water from WF wasp to PF wasp via the common stomach, despite the higher "rate" of water flux under larger *σ* values. That is, although larger *σ* values increase the rate of water transfer, they place a greater restriction on the range of possible water levels and might therefore never allow for the water level to reach levels where the rate of water transfer is very high. Note that linear resistance could still establish a sufficiently strong buffer while taking care to allow for freer water mediation and hence faster task completion. The finding that *σ* = 0 also implies that CS wasps accept/give the maximum amount of water possible for a given water level and therefore minimize the number of successes needed for complete emptying (for WF wasps) or filling (for PF wasps) at every water level. Thus, the number of successes that a WF wasp and PF wasp must complete for complete emptying and filling respectively when the common stomach is 50% full is 6, which is very close to the observed average values in wasp colonies [26]. Further, a linear resistance function may have also evolved because it might optimize mediation of water from WF wasps to PF wasps. Note also that the linear resistance function must be interpreted as the average resistance over the entire common stomach; it is possible that the resistance functions of individual CS wasps might not be linear and might also, in fact, vary among different CS wasps. This could allow for stronger regulation at the individual level while maintaining efficient mediation of water at the system level, thereby allowing the common stomach to perform both its buffering and regulating functions (Results Section 2).

Our third finding *K* = 0.988 ± 0.002 means that for every 100 failures observed (by any set of foragers, including WF wasps and PF wasps), 98 or 99 cases will be proceeded by another attempted interaction while the remaining 1 or 2 cases will have the forager aborting their current task. This finding agrees with empirical data of larger wasp colonies where task fidelity is high [7, 31] In these colonies foragers are very unlikely to prematurely abort their foraging cycle and do so only when the number of failures is very high, therefore these foragers could become specialists of the task and can benefit from their previous experience [39]. With the values we derived, our model correctly predicts the maximum number of successes made by a PF wasp (about 17), and we could pinpoint the average water level to be 44.7% (Results Section 3). Here, we comment that the leak that reduces the average water level to below 50% helps to keep the common stomach water level fluctuating and therefore maintains the mediation of water from WF wasps to PF wasps. Thus, we may conclude that the common stomach evolved to have a dual-purpose resistance function that, together with equal behavior of the CS wasps toward the WF and PF wasps, establishes a particular dependence of successes on the water level of the common stomach. Then, foragers evolved to have a low probability of aborting, just as in large real colonies [7], and this established a relationship between number of successes actually made and the average water level of the common stomach. The consequence is that on average the water level of the common stomach is just below 50%, and this ensures that the system operates with on average 5–6 successful interactions. This in turn improves information acquisition of colony needs [33, 34].

Having provided a possible biological grounding for our model parameters, we demonstrated in this study that it is possible to explain interaction-level trends as products of more fundamental individual-level behaviors. In particular, by detailing a mathematical description of the water transfer mechanism and the negative feedback mechanisms regulating the associated flux of water, we showed how certain fundamental parameters underlying these mechanisms can result in measurable trends that reflect the inner workings and symmetric structure of the common stomach. We were therefore able to uncover certain details about the common stomach based on how foragers interacted with it. These results elucidated the nature of several assumptions we used in previous models [28, 31, 30] and provided explanations of emergent properties of these models such as task fidelity, stabilized common stomach below 50% fullness and the existence of 5–7 successful interactions between foragers and common stomach wasps within a foraging cycle.

## Supporting Information

S1 File Data used to obtain the function between number of successful interactions and the total duration of these interactions in one foraging cycle. (XLSX)
